# Bulk and Surface Dual‐Modification for Stabilizing RuO_2_ Anode in 2 A cm^−2^ PEMWE Operation

**DOI:** 10.1002/adma.202521819

**Published:** 2026-03-06

**Authors:** Jiayi Tang, Zijun Fang, Yu‐Cheng Huang, Daqin Guan, Bin Chen, Zongping Shao

**Affiliations:** ^1^ Curtin Centre for Advanced Energy Materials and Technologies (CAEMT) Western Australian School of Mines (WASM) Curtin University Perth Western Australia Australia; ^2^ State Key Laboratory of Intelligent Construction and Healthy Operation and Maintenance of Deep Underground Engineering Guangdong Provincial Key Laboratory of Deep Earth Sciences and Geothermal Energy Exploitation and Utilization College of Civil and Transportation Engineering Shenzhen University Shenzhen China

**Keywords:** acidic oxygen evolution reaction, proton exchange membrane water electrolyzer, Ru‐oxide doping, catalyst surface engineering

## Abstract

RuO_2_ is a promising alternative to IrO_2_ for the oxygen evolution reaction (OER) in proton exchange membrane water electrolyzers (PEMWEs). However, to date, only Ir oxide or IrRu‐oxide based anodes have demonstrated possible stable operation at industrial‐relevant current densities of 2 A cm^−2^ for practical PEMWE applications. The poor durability of Ir‐free RuO_2_ anodes remains a major barrier to its practical use. While metal doping has been extensively explored to stabilize the Ru valence, emerging evidence suggests a multifactorial failure mechanism involving both physical and chemical degradation of RuO_2_ anodes. This underscores the need for strategies that stabilize both the catalyst bulk structure and reaction interface in OER. Here, we report a dual‐modification strategy combining bulk Cr substitution with Si surface modification to simultaneously enhance the intrinsic activity and stability of RuO_2_‐based anode in PEMWE. Cr doping modulates the Ru valence state in the bulk phase, promoting charge transfer while suppressing Ru overoxidation, whereas Si modification stabilizes the reaction interface by inhibiting catalyst reconstruction and protecting the Cr dopant. The resulting catalyst achieves stable operation in PEMWE at 2 A cm^−2^ and 1.65 V. This work provides new insights into the development of RuO_2_‐based catalysts and electrodes for PEMWEs.

## Graphical abstract

The high‐current‐density stability of the RuO_2_‐based anode in PEMWE could be taken care of by a bulk and surface dual modification strategy developed in this work, where Cr doping modulates the Ru valence state in the bulk phase, suppressing Ru overoxidation, whereas Si modification stabilizes the reaction interface by inhibiting catalyst reconstruction and protecting the Cr dopant.

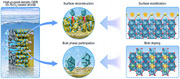

## 1 Introduction

Green hydrogen plays a vital role in decarbonizing energy‐intensive sectors such as fertilizer production and steel manufacturing. PEMWEs have received considerable attention for scaling green hydrogen production due to their high hydrogen production efficiency, product purity, and rapid dynamic response [1]. However, large‐scale deployment of PEMWEs is currently constrained by the high cost and limited availability of precious metal catalysts, particularly the heavy reliance on Ir for the anodic OER. To date, IrO_2_‐based catalysts remain the only materials capable of supporting stable PEMWE operation at industrially relevant current densities of 2 A cm^−2^, while ensuring cost effectiveness [2]. Complete replacement of Ir with Ru remains challenging, as up to now only Ir oxides or Ir‐stabilized Ru oxides have been demonstrated to operate stably at industrial current density of 2 A cm^−2^ [3, 4, 5].

Rutile RuO_2_ has emerged as a promising alternative to Ir‐based catalysts owing to its superior intrinsic OER activity and significantly lower cost. Nonetheless, most studies report poor long‐term stability of RuO_2_ under acidic and high‐current‐density PEMWE conditions, which remains a critical barrier to practical application. This instability has commonly been ascribed to alterations in the Ru electronic structure, including over‐oxidation of Ru sites and lattice oxygen participation through the lattice oxygen mechanism (LOM) [6, 7, 8]. As research moves ahead toward device‐level study of RuO_2_ anodes in PEMWEs, new evidence has revealed that intrinsic bulk stability can significantly extend durability. For instance, well‐crystallized, defect‐free RuO_2_ was found to sustain more than 500‐h operation at 1 A cm^−2^ with a slow decay rate of about 133 µV h^−^^1^ [9]. Beyond chemical state changes, physical degradation of anode catalyst layer has been recognized as another critical factor affecting the durability of PEMWEs [10]. These new insights suggest that there may be additional mechanisms beyond stabilizing the Ru valence that can be leveraged to prolong the stability of RuO_2_ electrodes at high current densities.

Recently, doping strategies have been widely adopted to modulate the Ru *d*‐band center [11], lattice strain [12, 13, 14], and/or oxygen vacancy concentration of RuO_2_ to benefit the acidic OER performance [15, 16]. However, these approaches typically focus on indiscriminate bulk doping, without considering whether such modifications are essential at the catalytic interface where OER occurs. Moreover, the tolerance of metal dopants under acidic OER is often overlooked, even though their instability may contribute to long‐term degradation. Given that the catalyst surface reconstruction genuinely drives the loss of reaction interface within electrode, catalyst surface engineering also deserves some attention to address the stability issue of RuO_2_.

In this work, for the first time, we report a dual‐modification strategy combining bulk Cr substitution with Si surface modification of RuO_2_ to simultaneously enhance both intrinsic activity and durability for acidic OER operating at industrial current density of 2 A cm^−2^. We applied a two‐step route for the synthesis of the bulk and surface dual‐modified catalyst (Si@CrRuO_2_). The resulting catalyst achieves 2 A cm^−2^ at 1.65 V, demonstrating its effectiveness as a PEMWE anode operating at an energy efficiency of around 75%. Long‐term operation at an industrially relevant current of 2 A cm^−2^ was enabled with a slow decay rate of only 64.4 µV h^−^^1^. On top of the performance metrics, this study highlights that RuO_2_ electrode degradation in high‐current‐density OER could be governed by multiple factors. While bulk electronic stabilization is essential, surface interface engineering is equally critical to achieve durable operation.

Both experimental and theoretical results show that the Cr incorporation into the Ru lattice modulates the Ru valence and stabilizes Ru valence state in the bulk phase, whereas Si surface modification further stabilizes the reaction interface by inhibiting the loss of lattice oxygen and catalyst reconstruction. In situ X‐ray absorption spectroscopy (XAS) conducted under electrolyzer operando voltages and current densities confirmed the dual stabilization mechanism. In addition, Si modification was evidenced to suppress both the Ru and the Cr oxidation under high‐current‐density OER, preserving the electron‐donation role of Cr and extending the operational potential and current density window for OER in PEMWE. Besides, Si surface engineering mitigates the rapid performance loss typically caused by accelerated surface reconstruction and interface degradation under intense reaction rates. By integrating surface modification with targeted bulk doping, this work provides a new design strategy for next‐generation RuO_2_ based catalysts for scalable, durable, and cost‐effective green hydrogen production through PEMWE.

## 2 Results and Discussion

### 2.1 Catalyst Characterizations and Electrochemical OER Performance

**FIGURE 1:**
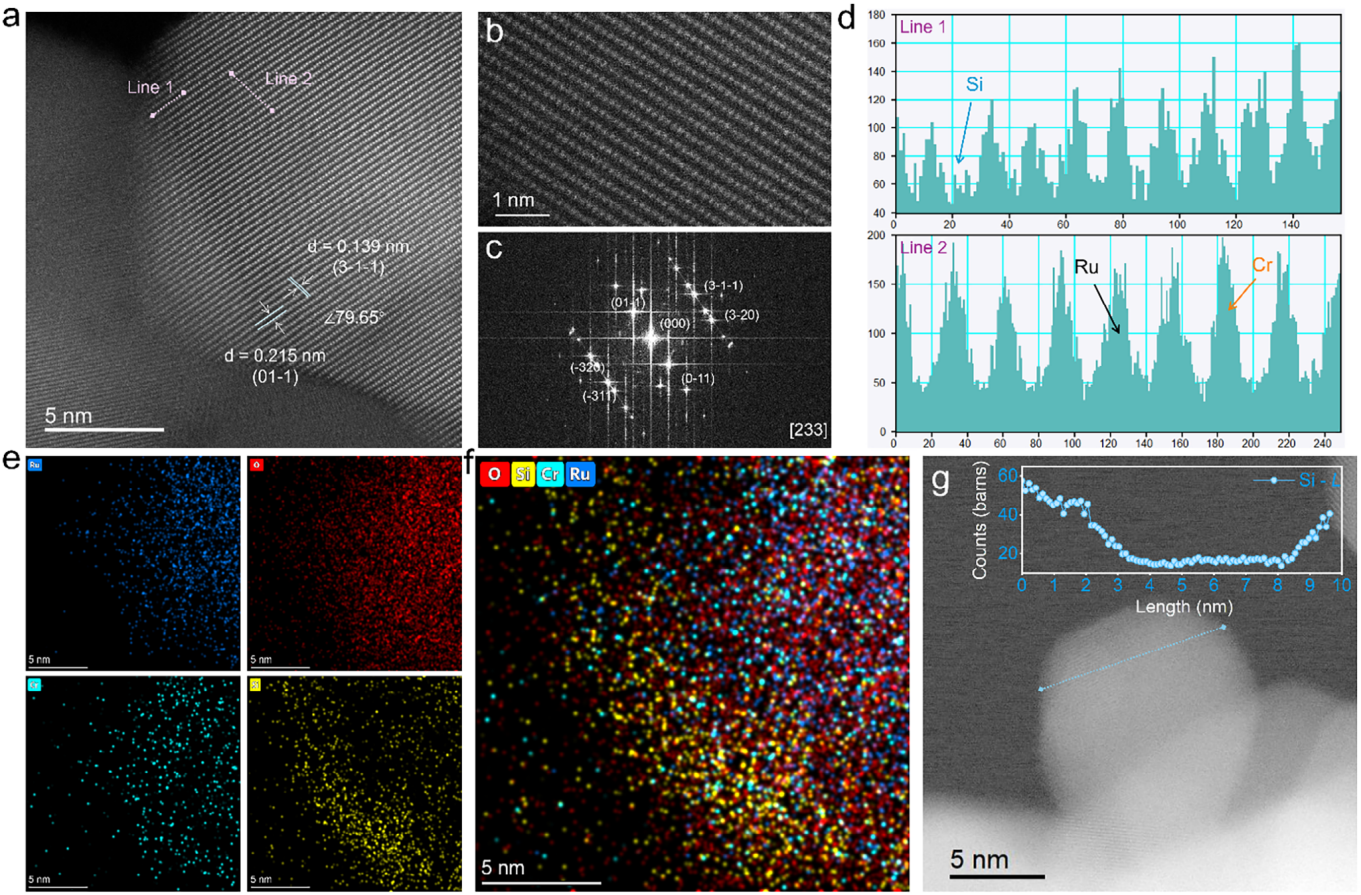
Catalyst design and characterizations. (a) HAADF‐STEM image of the synthesized Si@CrRuO_2_ nanoparticle. (b) High‐resolution HAADF‐STEM image showing the crystalline bulk structure. (c) The corresponding FFT pattern with indexed facets. (d) Line‐scan intensity profile extracted from the region highlighted by the pink lines. (e) EDS elemental mapping showing the individual distribution of Ru, O, Cr, and Si. (f) An overlay of the elemental maps. (g) EELS line‐scan profile of Si across a single particle.

The designed Si@CrRuO_2_ catalyst was synthesized through a two‐step process and characterized using spherical aberration–corrected transmission electron microscopy (TEM). TEM images and particle size analysis revealed that Cr doping reduced the average particle diameter to approximately 18.9 nm, compared to the synthesized undoped pure RuO_2_ (syn‐RuO_2_) presently as largely aggregated with average particle size of ∼ 48 nm under identical calcination conditions (Figures S1 and S2). Despite the smaller size, the Cr‐doped oxides (CrRuO_2_) maintained high crystallinity, with clear lattice fringes corresponding to the (110) and (101) planes of RuO_2_ (Figure S3). The high‐angle annular dark‐field scanning transmission electron microscopy (HAADF‐STEM) image (Figure 1a) of the synthesized Si@CrRuO_2_ particles shows aggregated, irregular nanoparticles incorporating both Cr doping and Si surface modification. A high‐resolution enlarged area (Figure 1b) reveals a highly crystalline particle with clearly resolved lattice fringes. The fast Fourier‐transformation (FFT) electron diffraction pattern (Figure 1c) derived from Figure 1a selected area is in [233] zone axis showing typical facets of rutile RuO_2_. Atomic line profile (Figure 1d) of the marked lines shows the possible Si modification at near the surface region and Cr doping in the bulk region, respectively. This two‐step synthesis strategy was deliberately designed to preserve bulk crystallinity to ensure good electronic conductivity, while introducing surface modification to enhance interfacial reaction performance. The elemental distribution of Si, Cr, Ru, and O analyzed by energy‐dispersive spectroscopy (EDS) mapping (Figure 1e,f; Figure S4) further confirmed the successful synthesis of Si@CrRuO_2_, showing Cr ions homogeneously distributed throughout the bulk phase, whereas Si was concentrated mainly at the surface. Electron energy loss spectroscopy (EELS) spectrum (Figure 1g) of Si collected across a single particle further confirms the enrichment of Si at the surface rather than penetrating into the bulk.

**FIGURE 2:**
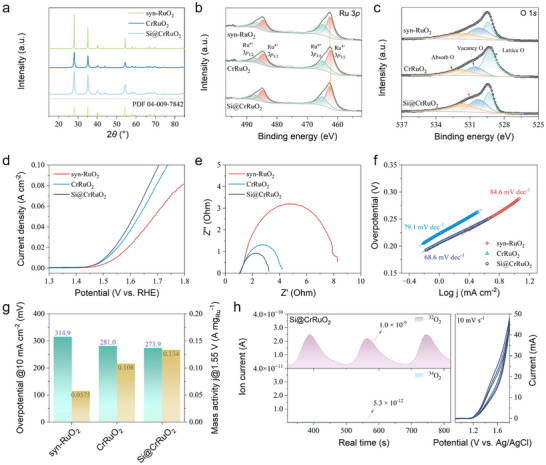
Structure and chemical state of the synthesized catalysts and electrocatalytic OER performance under RDE evaluation. (a) XRD patterns of the synthesized Ru oxides powder. High‐resolution XPS fine spectra of (b) Ru 3*p* orbital and (c) O 1*s* orbital with deconvoluted peaks. (d) Geometry‐normalized LSV curves obtained in 0.5 m H_2_SO_4_ electrolyte. (e) Nyquist plots from EIS measurements. (f) Tafel plots derived from steady‐state polarization curves in the activation zone. (g) Bar plot comparison of the OER overpotentials and mass activities. (h) DEMS signals of ^32^O_2_ and ^34^O_2_ for ^18^O‐labeled Si@CrRuO_2_ catalyst (with baseline subtracted) and the corresponding CV cycles.

Both the surface properties and bulk‐phase structure contribute to the electrocatalytic OER performance. While the electrochemical reaction occurs at the catalyst interface, efficient electron transport must proceed through the catalyst bulk. The rutile RuO_2_ structure plays a key role in this behavior due to its excellent intrinsic electronic conductivity [17]. X‐ray diffraction (XRD) analysis (Figure 2a) confirmed that all synthesized Ru oxides predominantly exhibited the rutile phase of RuO_2_, with no detectable secondary phases or segregation. Cr^3+^ ions, with an ionic radius (∼0.62 Å) comparable to that of Ru^4+^ (∼0.63 Å), are likely to substitute for Ru sites within the lattice, thereby forming CrRuO_2_. Inductively coupled plasma optical emission spectroscopy (ICP‐OES) tests identified the successful doping of Cr even at relatively low atomic ratios (Table S1). Compared to syn‐RuO_2_, Cr doping led to reduced diffraction peak intensity and slight peak broadening, suggesting suppressed grain growth and smaller crystallite size, consistent with the TEM observations. The Si@CrRuO_2_ catalyst retained similar characteristic peak positions to those of CrRuO_2_ and syn‐RuO_2_, but exhibited increased peak intensity, indicating that the post‐calcination treatment following surface impregnation with the Si precursor promoted mild particle aggregation.

The surface chemical states and local electronic structures were further examined through X‐ray photoelectron spectroscopy (XPS) and synchrotron‐based XAS. The survey spectra confirmed the characteristic orbital features of the synthesized Ru oxides (Figure S5). To avoid overlap between the Ru 3*d* and C 1*s* signals, high‐resolution Ru 3*p* spectra were analyzed instead, as shown in Figure 2b. The representative Ru 3*p_3/2_* peak can be deconvoluted into a primary component corresponding to Ru^4+^ at 462.44 eV and a satellite peak attributed to partially reduced Ru^n+^ species at 464.69 eV. Upon Cr doping, the Ru^4+^ 3*p_3/2_* peak shifted slightly to a lower binding energy (462.30 eV), suggesting electron donation from Cr^3+^ substitution at Ru sites, which slightly reduces the Ru valence and may protect Ru from over‐oxidation. A similar trend was observed in the Ru^4+^ 3*p_1/2_* peak, which shifted from 484.54 to 484.48 eV. This was further supported by the O 1*s* spectra (Figure 2c), where the lattice oxygen peak shifted to a lower binding energy (529.27 eV) compared with syn‐RuO_2_ (529.43 eV). Additional surface Si modification caused a slight positive shift of the Ru^4+^ 3*p_3/2_* peak to 462.47 eV, reflecting the formation of Si─O─Ru bonds that increase the surface oxidation state of Ru due to the strong electronegativity of Si^4+^. Moreover, the O 1*s* spectra displayed a pronounced shoulder peak near 531.00 eV, indicative of an active surface with oxygen vacancies generated from Si modification. Ru *K*‐edge X‐ray absorption near‐edge structure (XANES) spectra (Figure S6) further confirmed the XPS observations. The absorption edge of CrRuO_2_ slightly shifted toward lower energy, indicating partial reduction of Ru^4+^ with the electronic donation effect from Cr^3+^. In contrast, the Si@CrRuO_2_ exhibited a minor positive edge shift, corresponding to the formation of Si─O─Ru bonds on the surface. Reference spectra of Ru foil and RuO_2_ were used for edge calibration and valence comparison. *R*‐space fitting of the FT‐EXAFS (Figure S7 and Table S2) showed a reduced coordination number after Cr doping and Si modification. In addition, a shortened Ru─O bond length was observed, which also indicates the formation of Si─O─Ru bonds due to the high electronegativity and small ionic radius of Si.

The influence of Cr doping and Si modification on the intrinsic OER activity was evaluated using a rotating disk electrode (RDE) system. As shown in Figure 2d,g, Cr doping significantly enhanced catalytic activity, reducing the onset potential to 273.9 mV at 10 mA cm^−2^ compared to 314.9 mV for syn‐RuO_2_. In contrast, while Si modification did not substantially reduce the overpotential, it further improved the OER performance at higher current densities. This improvement is supported by the Nyquist plots from electrochemical impedance spectroscopy (EIS) measurements (Figure 2e), which exhibited a substantial decrease of the charge‐transfer resistance (R_ct_) with Cr doping and Si modification. The enhanced OER reaction kinetics were further confirmed by the Tafel slopes (Figure 2f), where Si@CrRuO_2_ exhibited a value of 68.6 mV dec^−^^1^, lower than that of CrRuO_2_ (79.1 mV dec^−^^1^) and syn‐RuO_2_ (84.6 mV dec^−^^1^). The synergistic effect of Cr doping and Si modification resulted in a superior mass activity of 0.134 A mg_Ru_^−1^ for the Si@CrRuO_2_. Electrochemical surface area (ECSA) measurements indicate an increased active surface area after Si modification (Figures S8 and S9). In addition, in situ differential electrochemical mass spectrometry (DEMS) measurements (Figure 2h) of ^18^O‐labeled catalysts revealed no evidence of lattice oxygen participation during OER. The main product detected was ^32^O_2_ over multiple cyclic voltammetry (CV) cycles, and the performance remained stable. This indicates that the improved OER performance arises from surface activation induced by Si modification and bulk lattice oxygen stabilization by Cr doping, rather than lattice oxygen involvement.

Catalyst stability was evaluated under RDE conditions following a typical protocol at a constant current density of 10 mA cm^−^^2^ (Figure S10). While RDE testing can mimic the acidic OER environment, its inherent rotation effects and limited mass transport make it an imperfect analogue for real PEMWE stability evaluation. Nonetheless, for comparison, the optimized Si@CrRuO_2_ catalyst exhibited significantly improved durability, maintaining stable operation for over 250 h, whereas syn‐RuO_2_ failed after approximately 80 h.

To highlight the advantage of Si surface modification over bulk co‐doping, we studied the electrochemical performance of Si doped RuO_2_ (marked as SiRuO_2_), where Si was incorporated into the bulk phase through a one‐step calcination process, similar to the synthesis of CrRuO_2_. As shown in Figures S11 and S12, we found that significant Si incorporation into the bulk rather than surface localization could exhibit diminished OER activity. This decline could be attributed to the non‐metallic nature of Si, which, when incorporated into the bulk lattice of RuO_2_, could break in the electron conduction pathways, increasing the bulk resistivity of the catalyst. As reflected by the Nyquist plots, an increased R_ct_ was observed for SiRuO_2_ during OER catalysis. The impeded electron transport becomes particularly pronounced at high current densities, where efficient and rapid electron mobility is critical to sustain the oxygen evolution reaction. These results indicate that bulk co‐doping with Si may counteract the beneficial effects of Cr doping.

This observation is in line with many reported studies on RuO_2_ doping, which generally maintain dopant levels below 10 at.% but seldom explicitly emphasize the detrimental effects of further increasing dopant concentrations [18]. Excessive distortion of the rutile structure can adversely impact catalytic performance, making bulk doping a double‐edged strategy. Even for Si surface modification, the modification level was deliberately controlled, as higher Si content was observed with some surface accumulation and excessive coverage of the Ru oxide (Figure S13). These insights motivated the dual‐modification design of Si@CrRuO_2_ in this work, which simultaneously taking advantage of the catalyst surface and bulk properties to benefit the OER process.

To further investigate the effects of doping and surface modification levels, we systematically varied the concentrations of Cr and Si to produce a series of Si_x_@Cr_y_RuO_2_ catalysts (Figures S14 and S15). Linear sweep voltammetry (LSV) curves and comparative analyses of overpotentials and mass activities revealed that increasing the Cr doping level beyond 2 at.% did not lead to significantly further reductions in overpotential. This provides insight into the role of Cr^3+^ substitution in modulating the Ru *d*‐band centre, thereby enhancing charge transfer through the bulk at even low doping levels. However, excessive Cr doping slightly decreased performance, likely due to surface accumulation of Cr oxide that hindered the exposure of Ru active sites. Similarly, Si surface modification controlled below 8 at.% was demonstrated to be beneficial, whereas higher Si content may lead to the formation of Si oxide that partially blocks the active surface and limits access to catalytic sites. Based on these findings, Si@CrRuO_2_ with 8 at.% Si and 2 at.% Cr was selected as the representative catalyst for demonstrating PEMWE performance and conducting in situ XAS investigations.

**FIGURE 3:**
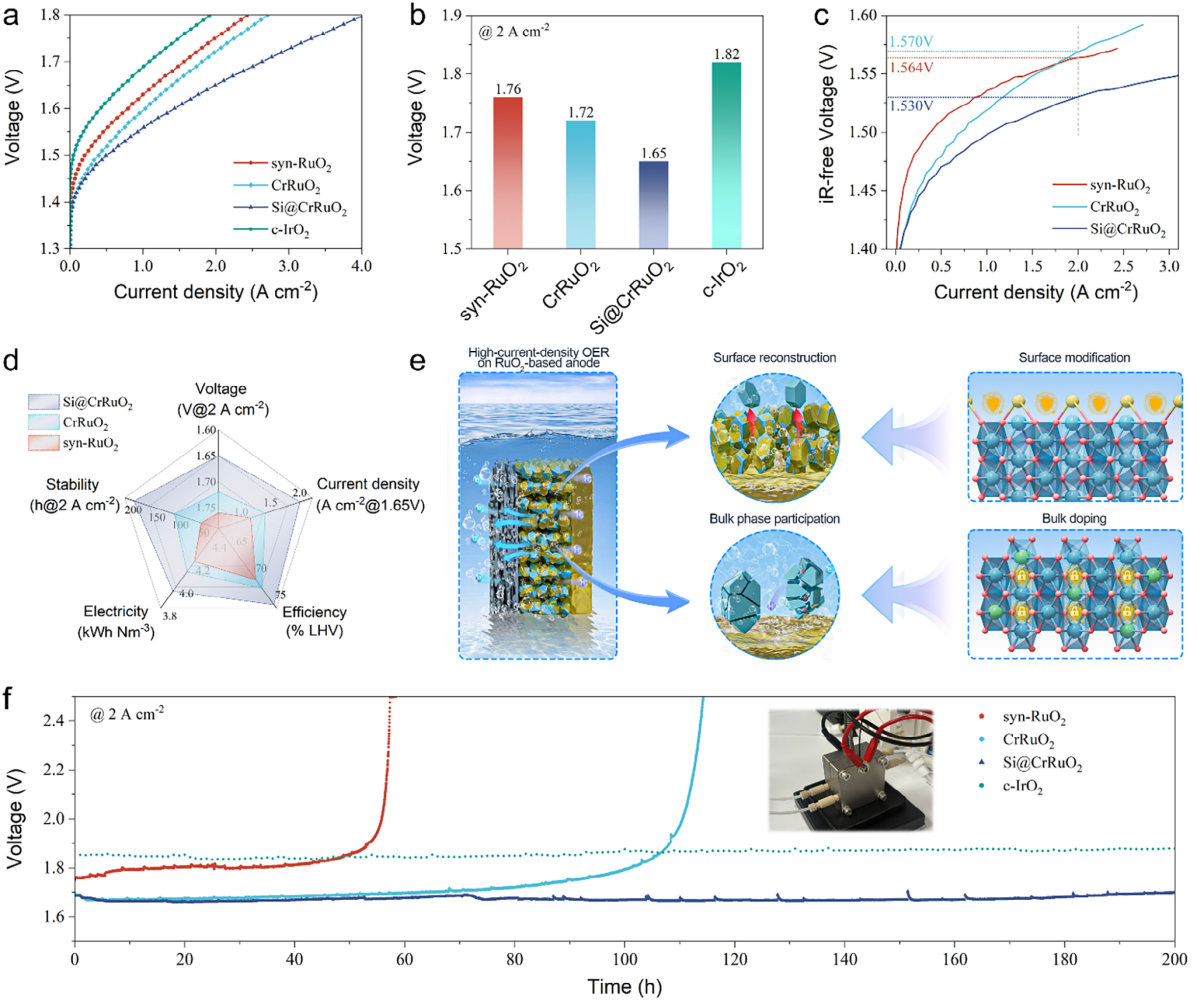
PEMWE performance with the designed catalysts and mechanism illustration. (a) PEMWE polarization curves obtained at 80°C with deionized water feed. Pt/C was used as the cathode catalyst, while the synthesized Ru‐oxide catalysts and the benchmark commercial IrO_2_ (c‐IrO_2_) served as the anodes. (b) A bar plot comparison of the voltages for operating the PEMWE at 2 A cm^−2^ with various anodes. (c) Performance curves with Ohmic loss correction applied (*iR*‐free). (d) Radar diagram comparing key PEMWE performance metrics of the synthesized Ru‐oxide catalysts. (e) Illustration of the instability mechanism of Ru oxide‐based anode at high‐current‐density operation and the dual‐modification stabilization strategy. (f) Stability of the PEMWEs with different anodes at 2 A cm^−^^2^, and the inset photo shows the 2 × 2 cm^2^ electrolyzer in testing.

To demonstrate the practical potential of the designed catalyst for application, the electrocatalytic OER performance of Si@CrRuO_2_ was thoroughly evaluated as the anode catalyst under PEMWE operating conditions. For a fair and reliable performance comparison, all electrolyzer assemblies were fabricated under identical conditions using a catalyst‐coated membrane (CCM) configuration with the Nafion NR212 membrane (∼50 µm). The anode Ru loading was fixed at 1.0 mg cm^−2^ for the tested catalysts, while the Pt/C cathode with a fixed Pt loading of 1.0 mg cm^−2^ was used in all fabrications. The assembled PEMWE with an active area of 2 × 2 cm^2^ was operated at 80 °C with pure water supplied to both the cathode and anode. The PEMWE performance curves in Figure 3a and Figure S16 with error bars exhibit that the Si@CrRuO_2_ anode delivered an outstanding current density of 4.03 A cm^−^^2^ at 1.8 V, significantly outperforming the CrRuO_2_ anode for achieving 2.71 A cm^−^^2^ and the syn‐RuO_2_ anode (2.43 A cm^−^^2^) under the same voltage. At the industrially relevant current density of 2 A cm^−^^2^, the designed Si@CrRuO_2_ anode exhibited a clear efficiency advantage, requiring an applied voltage of only 1.65 V (Figure 3b), in comparison with the c‐IrO_2_ that was driven by 1.82 V.

To better compare the intrinsic catalytic performance of the anode catalysts in PEMWE, and to exclude the influence of performance gains arising from an optimized electrolyzer assembly (which typically presents lower ohmic resistance), the *iR*‐corrected polarization curves were evaluated (Figure 3c). The high‐frequency resistance (HFR) values were extracted from the original Nyquist plots (Figure S17) at the intersection with the real impedance axis and were summarized in Table S3. The *iR*‐corrected polarization curves further demonstrate that Cr doping effectively enhances the intrinsic catalytic activity of the anode by lowering the onset potential, while Si modification primarily supports improved high‐current‐density operation.

Accelerated durability testing was performed by operating the PEMWE at 2 A cm^−^^2^. It is increasingly recognized that electrode performance degradation under device‐level high current density operation is governed by multiple factors, as illustrated in Figure 3e. Internally, the instability in valence state and lattice structure of the catalyst under the acidic and oxidative OER environment can lead to particle pulverization and eventual collapse of the electrode structure. On the surface, combined with water coordination and gas evolution, the severe catalytic reaction at high current densities can drive catalyst surface reconstruction, resulting in the loss of the active interface or catalyst detachment. As shown in Figure 3f, the unmodified syn‐RuO_2_ exhibited the fastest degradation, failing within 60 h of operation. Some previous findings suggest that crystalline RuO_2_ with a robust lattice demonstrated considerable stability [9]; however, Ru oxides synthesized under laboratory air calcination conditions typically feature lower durability than commercial crystalline RuO_2_. This poor stability is likely to be associated with the large specific surface area of the synthesized catalysts, which is accompanied by a high density of surface defects generated during conventional synthesis. Incorporation of Cr dopants into the RuO_2_ lattice promotes charge transfer to Ru sites, which may induce the formation of random lattice oxygen vacancies. However, owing to the similar ionic radii Cr^3+^ (0.62 Å) compared to the Ru^4+^ (0.63 Å), the extent of lattice oxygen vacancy generation is relatively limited. This conclusion is supported by electron paramagnetic resonance (EPR) analysis, as shown in Figure S18. The signal at g = 2.003, which can be attributed to the unpaired electrons associated with oxygen vacancies, shows only a slight increase for CrRuO_2_ compared with syn‐RuO_2_, while the overall signal intensity remains relatively weak. Subsequent Si surface modification further compensates surface defects. When combined with the observed stability performance, these results indicate that Cr dopants primarily act to protect Ru sites from over‐oxidation and to stabilize the Ru─O framework within the bulk phase. As a consequence, the PEMWE employing a CrRuO_2_ anode achieved an extended operational lifetime. Nevertheless, under industrially relevant high current densities, failure still occurred after approximately 110 h of operation, meaning that protecting the Ru catalytic sites from over oxidation may not be sufficient to sustain long‐term and intense operation.

In contrast, over 200 h of continuous operation, the Si@CrRuO_2_‐based PEMWE exhibited a small performance decay rate of 64.4 µV h^−^^1^ for the operation at 2 A cm^−2^, which is comparable to the trend observed with the commercial IrO_2_ (Figure 3f), and even more stable than the best‐performing catalysts typically reported at the operating current density of 1 and 2 A cm^−2^ (Table S4). Even higher stability was observed for the operation at 1 A cm^−2^, with a decay rate of only 44.4 µV h^−^^1^ during a 450‐h long‐term operation (Figure S19). The catalyst morphology and composition remained intact after the stability test (Figure S20). Inductively coupled plasma mass spectroscopy (ICP‐MS) was used to evaluate the possible dissolution of the catalysts during high–current‐density operation. As shown in Table S5, only ppb‐level negligible Ru dissolution was detected, with minimal Cr and Si presence below the precise threshold limit. The dissolution amount accounts for negligible catalyst loss in the electrode, thus is less likely to cause the performance degradation. These results suggest that the failure of Ru‐oxide anodes is not driven by extensive Ru dissolution but is more likely to be associated with the catalyst surface reconstruction. The enhanced stability observed with Si@CrRuO_2_‐based PEMWE demonstrates that Si surface modification can effectively inhibit the reaction interface loss by suppressing surface reconstruction while avoiding bulk distortion.

Figure 3d summarizes the key PEMWE performance metrics, underscoring the practical value of applying this dual‐modified catalyst in real‐world PEMWE systems. Operating PEMWE at 2 A cm^−2^ with the Si@CrRuO_2_ anode required a low voltage of only 1.65 V, corresponding to an energy consumption of 3.95 kWh Nm^−3^ and an energy efficiency of 75.3%.

### 2.2 Study of the Stability Mechanism Behind Si and Cr Dual Modification

**FIGURE 4:**
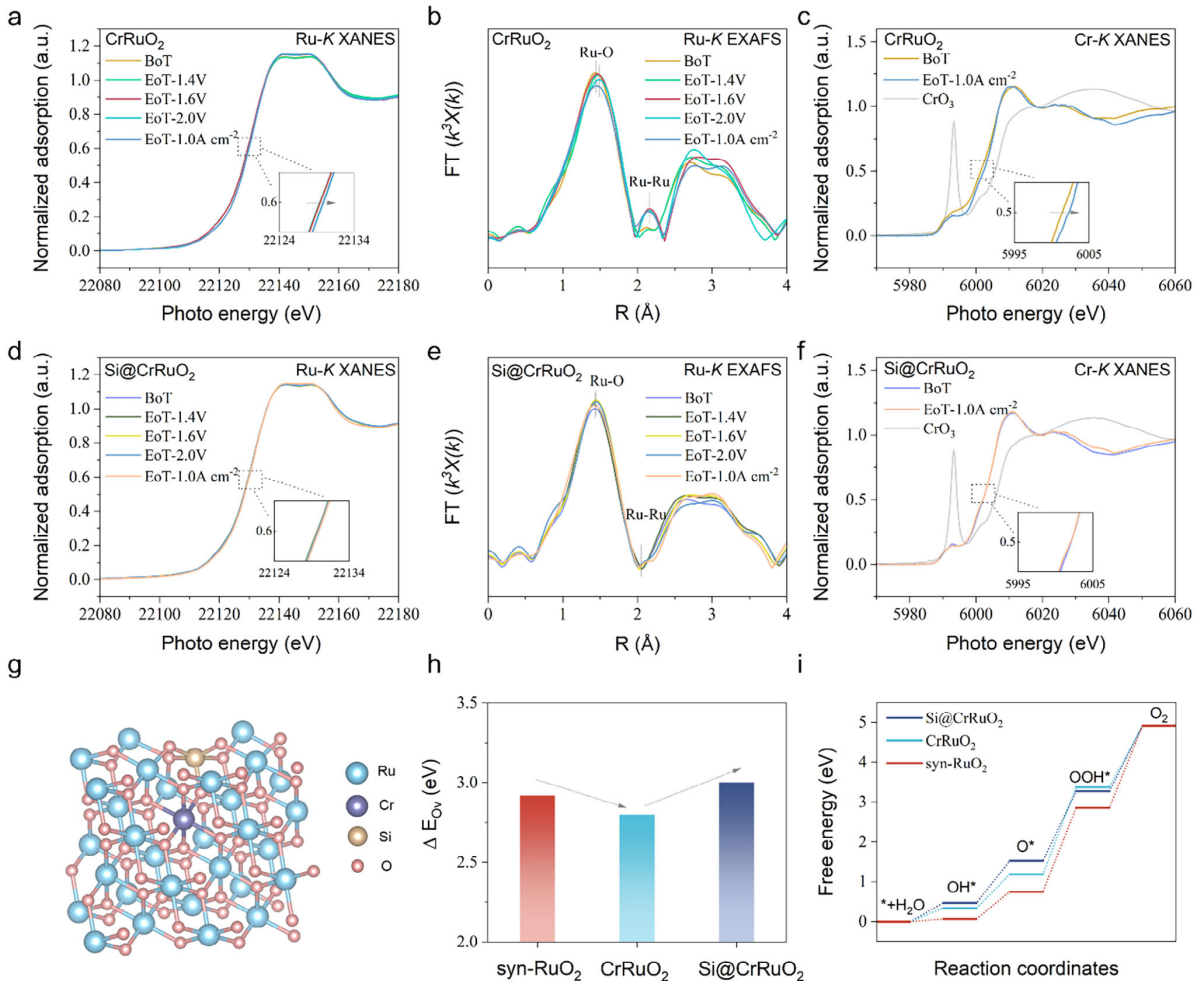
In situ XAS measurements under PEMWE relevant voltages and current densities. (a) Ru *K*‐edge XANES spectra, (b) Fourier‐transformed Ru *K*‐edge EXAFS spectra, and (c) Cr *K*‐edge XANES spectra of the synthesized CrRuO_2_ before and after the OER reaction. (d) Ru *K*‐edge XANES spectra, (e) EXAFS spectra, and (f) Cr *K*‐edge XANES spectra of the Si modified Si@CrRuO_2_ before and after the OER reaction. BoT is a short term of begin of test, and EoT is end of test. (g) Structural model of the designed Si@CrRuO_2_ catalyst featuring Cr bulk substitution and Si surface modification. (h) Comparison of the calculated vacancy formation energies for lattice oxygen. (i) Free‐energy profiles of the four‐step OER pathway on the synthesized Ru oxides.

To gain deeper insight into the enhanced OER performance arising from both Cr doping and Si surface modification, in situ XAS measurements were performed under operating voltages and current densities relevant to PEMWE (Figure 4; Figures S21 and S22). Cr is an interesting dopant that has been reported in several studies to enhance both OER kinetics and stability [19, 20, 21, 22]. However, the endurance of Cr itself under acidic OER conditions has received little attention in existing studies. Determining whether a metal dopant acts as a self‐sacrificial protector of Ru is essential for understanding the durability limits of the doped RuO_2_ catalysts. Ru *K*‐edge XANES spectra were collected for CrRuO_2_ at an operating potential range from 1.4 to 2.0 V (after holding each for 60 min), as well as under a current density of 1.0 A cm^−^^2^. At this current density, the corresponding applied voltage was around 3.0 V. Across the potential range of 1.4–2.0 V, Figure 4a shows nearly invariant edge positions, indicating that the average Ru oxidation state remained close to +4. The white‐line intensity also exhibits negligible variation, confirming the electronic stabilization provided by Cr^3+^ substitution. However, the operation under 1.0 A cm^−^^2^ was observed with a small edge shift toward higher energy adsorption, consistent with a slight increase in the apparent Ru valence. Nevertheless, the average oxidation state of Ru remained close to +4, suggesting minimal net oxidation of Ru sites even at high current density and elevated operating voltages.

In contrast to the relatively stable average valence, notable changes were observed in the local coordination environment of Ru. The Fourier‐transformed Ru *K*‐edge extended X‐ray absorption fine structure (EXAFS) spectra (Figure 4b) display a slight radial contraction and amplitude decrease of the first Ru─O shell (∼1.9 Å) above 1.6 V, reflecting partial oxygen vacancy formation and decreased coordination. Simultaneously, a weak Ru─Ru contribution (∼3.1 Å) emerged, suggesting local reconstruction and partial disruption of Ru─O─Cr linkages under anodic bias. After operation at 1.0 A cm^−^^2^ for 60 min, partial recovery of the Ru─O shell position was detected, further supporting a restructuring process under load. The Cr *K*‐edge spectra (Figure 4c) exhibit a discernible edge shift toward higher energy relative to the oxidized Cr oxide (CrO_3_ as referenced), indicating oxidation of Cr^3+^ to a higher valence state during high‐current operation. This observation suggests a self‐oxidative sacrificial behavior of Cr dopant, which temporarily stabilizes the Ru^4+^ lattice but may induce local structural disorder upon severe OER process. Taken together with the electrolyzer performance results shown in Figure 3, these findings suggest that while metal doping can act as an electron donor to suppress Ru valence changes by tuning the electronic structure, the inherent susceptibility of dopants such as Cr to oxidation may trigger structural reconstruction. As a result, stability remains challenged under high‐current operation where more active sites are engaged.

As shown in Figure 4d and Figure S22, upon Si surface modification, the Ru *K*‐edge XANES spectra of Si@CrRuO_2_ exhibit nearly invariant edge positions and white‐line intensities over the entire potential range and at 1.0 A cm^−^^2^ operation as well, demonstrating a highly stabilized Ru^4+^ valence. Corresponding Ru *K*‐edge EXAFS spectra (Figure 4e) reveal that the first Ru─O shell (∼1.9 Å) remained at a constant radial distance with only minor amplitude attenuation, while the Ru─Ru scattering feature (∼3.1 Å) was strongly suppressed. These results indicate that Si modification effectively inhibits lattice rearrangement and prevents the surface reconstruction observed with Cr doping alone. Importantly, this surface‐specific modification by Si did not compromise the bulk‐phase electronic conductivity of the catalyst. In addition, Si modification was observed to prevent Cr oxidation under high‐current‐density OER, as evidenced by the absence of a Cr *K*‐edge shift in XANES spectra (Figure 4f). This protection allowed Cr to maintain its electron‐donation role, thereby extending the operational stability window to higher potentials and current densities.

Density functional theory (DFT) calculations were performed to provide theoretical insight into the enhanced OER kinetics and the stabilized active sites induced by Cr doping and Si modification. Structural models of syn‐RuO_2_, CrRuO_2_, and Si@CrRuO_2_ were constructed based on XRD profiles of the synthesized catalyst powders (Figure 4g; Figures S23 and S24). The highly active (101) facet of rutile RuO_2_, which typically exposes coordinatively unsaturated Ru sites, was selected as the representative surface for the calculations. The oxygen vacancy (O_v_) formation energy was first calculated to evaluate the strength of Ru─O bonding and the tendency toward surface reconstruction. As shown in Figure 4h, CrRuO_2_ exhibits a reduced O_v_ formation energy of 2.799 eV compared to pristine syn‐RuO_2_, which shows a relatively higher value of approximately 2.919 eV. This result suggests that Cr substitution promotes charge redistribution to Ru sites, facilitating O_v_ formation. Although pristine syn‐RuO_2_ shows a higher vacancy formation energy, experimental results demonstrate significantly improved kinetics and stability upon Cr doping, indicating that the O_v_ generated by Cr substitution is not detrimental and can be stabilized, which has also been demonstrated in other works studying the Cr doping mechanism [19]. However, the instability of RuO_2_ in acidic OER does not solely originate from the valence alteration but also from the loss of the effective reaction interface, as discussed above. With additional Si modification, surface lattice oxygen is further stabilized, as reflected by an increased O_v_ formation energy increased to 3.001 eV, effectively inhibiting surface reconstruction during the OER.

The four‐step OER reaction pathway was further evaluated (Figure 4i). The formation of the OOH^*^ intermediate from O^*^ was identified as the rate‐determining step for all synthesized Ru oxide catalysts, consistent with the adsorbate evolution mechanism (AEM). Pristine syn‐RuO_2_ exhibits rapid initial proton–electron transfer and fast population of OH^*^ species, which leads to a high energy barrier for OOH^*^ of 2.108 eV. The resulting buildup of early intermediates is likely to promote surface reconstruction. Upon Cr doping, the free‐energy barriers of the elementary OER steps become more evenly distributed, indicating a more balanced proton–electron transfer process across the reaction pathway. Notably, with additional Si modification, the energy barrier for OOH^*^ formation is significantly reduced to 1.747 eV for Si@CrRuO_2_. Meanwhile, the moderated OH^*^ deprotonation barrier (1.052 eV) suggests suppressed O^*^ accumulation, which alleviates surface amorphization during OER and mitigates Ru leaching associated with irreversible surface reconstruction. As a result, both enhanced OER kinetics and improved catalyst durability are theoretically validated.

### 2.3 Further Elucidating the Role of Si Modification

**FIGURE 5:**
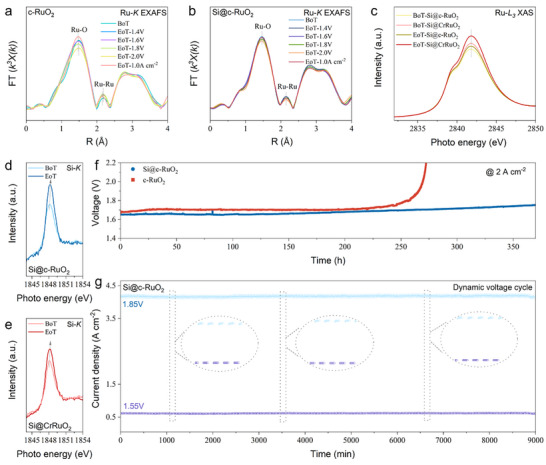
In situ XAS measurements and stability evaluation of Si‐modified commercial RuO_2_ (Si@c‐RuO_2_). Fourier‐transformed Ru *K*‐edge EXAFS spectra of (a) the commercial RuO_2_ without modification (c‐RuO_2_), and (b) Si‐modified Si@c‐RuO_2_. (c) Ru *L_3_*‐edge XAS spectra of the Si@c‐RuO_2_ and the synthesized Si@CrRuO_2_ before and after OER at 1.0 A cm^−^^2^. Si *K*‐edge XANES spectra of (d) the Si@c‐RuO_2_, and (e) the Si@CrRuO_2_ before and after the reaction. (f) PEMWE stability comparison of the c‐RuO_2_ and the Si@c‐RuO_2_ at 2 A cm^−^^2^. (g) Stability evaluation of the Si@c‐RuO_2_ under dynamic cycling between 1.55 and 1.85 V with 15 min potential holds at each step for 9000 min.

To further elucidate the effect of Si modification, we modified commercial RuO_2_ (c‐RuO_2_) and examined how bare Si surface modification contributes to reaction interface stabilization. In our previous work, highly crystalline RuO_2_ with a robust bulk structure showed promising PEMWE performance, but long‐term operation under 1.0 A cm^−2^ led to gradual performance decay, which is likely due to catalyst surface reconstruction [9]. In situ Ru *K*‐edge XANES spectra and EXAFS spectra (Figure 5a; Figure S25), collected under various operating voltages and current densities, revealed that c‐RuO_2_ remained stable at relatively low voltages (<1.6 V), as evidenced by the consistent peak positions of both the first Ru─O shell and the second Ru─Ru shell. However, operation above 1.8 V led to a slight shift and amplitude decrease in the first Ru─O shell, accompanied by a pronounced negative shift in the Ru─Ru scattering path under 1.0 A cm^−^^2^ operation. These results indicate that over coordination with water reactants on Ru sites may accelerate surface reconstruction when operated at high current densities and corresponding high voltages.

In contrast, the Ru *K*‐edge EXAFS spectra (Figure 5b) of the Si‐modified counterpart, Si@c‐RuO_2_, show slightly increased white‐line intensity after OER operation, indicative of enhanced Ru 4d–O 2p hybridization due to stronger adsorption of oxo intermediates [18, 23]. Increased electrode surface hydrophilicity was observed with Si modification (Figure S26), which indicates possible enhanced water coordination [24]. Additional evidence came from the Ru *L_3_*‐edge spectra (Figure 5c) of both Si@CrRuO_2_ and Si@cRuO_2_, where the increased white‐line intensity suggests enhanced coordination of water molecules or oxo‐intermediates with the surface during the OER [25, 26]. Importantly, no significant peak position change was observed, indicating that the Ru orbital occupation state remained largely intact. Although partial Cr loss may occur during OER, the presence of Si appears to stabilize the Ru electronic structure. These findings indicate that the role of Si modification here is not to predominantly enhance activity (Figure S27), but to maintain the reaction interface by mitigating the surface reconstruction induced by intense water coordination over efficient operation. This can be particularly meaningful for practical application, as RuO_2_ already exhibits superior intrinsic activity compared to IrO_2_, sufficient to support high‐efficiency PEMWE operation if further stabilized.

To further validate the benefits of Si surface modification, stability testing was conducted under PEMWE operation at 2 A cm^−2^ (Figure 5f). Over a 350‐h testing period, the Si@c‐RuO_2_ anode exhibited a slow and gradual voltage increase at a rate of 0.27 mV h^−1^. This contrasts sharply with the sudden, accelerated failure observed for the c‐RuO_2_ anode after an operation of 200 h, which is also an instability profile commonly reported with synthesized Ru‐oxide catalysts in acidic OER [12, 13, 27]. These results indicate that Si surface modification mitigates the rapid performance drop typically caused by the abrupt loss of the reaction interface through accelerated Ru surface reconstruction to a certain extent under high‐current‐density operation.

However, although catastrophic failure was avoided, the sluggish voltage rise still observed during long‐term operation suggests that Si modification alone does not fully eliminate degradation. Further stabilization of the catalyst bulk, together with more efficient charge transfer and mass transport, is required to mitigate physical degradation in addition to chemical‐state degradation. Recent studies have likewise shown that RuO_2_ degradation in acidic OER can be predominantly governed by water/proton‐associated interfacial reactions rather than progressive oxidation of Ru sites [28]. Complementary Si *K*‐edge XANES spectra (Figure 5d,e) revealed a subtle positive edge shift relative to the SiO_2_ reference, suggesting increased polarization of Si─O bonds through stronger interactions with adsorbed water molecules for both the Si@CrRuO_2_ and Si@c‐RuO_2_ after reaction [29, 30]. To test whether controlling interfacial water coordination could enhance stability, we applied a dynamic voltage protocol alternating between 1.55 and 1.85 V. This approach aims to regulate water adsorption and desorption during operation. As shown in Figure 5g, Si@c‐RuO_2_ maintained stable current output for over 150 h under this dynamic mode, demonstrating that a stabilized reaction interface can be achieved when Si surface modification is combined with operational strategies that regulate water coordination.

## 3 Conclusion

Here, we report the design of a bulk and surface dual‐modified catalyst, Si@CrRuO_2_ for acidic OER operating at an industrial‐relevant current density of 2 A cm^−2^. The results highlight the synergistic enhancement achieved through bulk doping and surface engineering, enabling stable PEMWE performance at high efficiency with Ir‐free anode. The conclusions of this work emphasize the critical role of catalyst surface engineering and dopant protection in the design of RuO_2_‐based catalysts. Inhibiting surface reconstruction is shown to be as important as stabilizing Ru valence. Both chemical and physical degradation of the anode should be addressed in developing next‐generation RuO_2_‐based catalysts aiming to replace Ir.

## Author Contributions

J.T. designed and performed the electrolyzer experiments, analyzed the data, and wrote the original manuscript. Z.F. synthesized the catalysts and performed the RDE tests. Y.C.H. and D.G. performed the synchrotron tests. B.C. coordinated the spherical‐aberration TEM characterisations. Z.S. supervised the project.

## Conflicts of Interest

The authors declare no conflicts of interest.

## Supporting information




**Supporting File**: adma72746‐sup‐0001‐SuppMat.docx.

## Data Availability

The main data supporting the findings of this study are available within the published article and its Supplementary Information. Additional data are available from the corresponding author on request.
