# Supplementary material for: Bulk and Surface Dual‐Modification for Stabilizing RuO2 Anode in 2 A cm−2 PEMWE Operation
Source: Adv Mater. 2026 Mar 6;38(19):e21819. doi: 10.1002/adma.202521819 (PMC13040509; doi:10.1002/adma.202521819)
Supplement: Supplementary file 1 — Supporting File: adma72746‐sup‐0001‐SuppMat.docx. [file ADMA-38-e21819-s001.docx]

**Supplementary Information**

**Title: Bulk and Surface Dual-Modification for Stabilizing RuO_2_ Anode in 2 A cm^–2^ PEMWE Operation**

Jiayi Tang^1^, Zijun Fang^1^, Yu-Cheng Huang^1^, Daqin Guan^1^, Bin Chen^2^, and Zongping Shao^1^*

^1^Curtin Centre for Advanced Energy Materials and Technologies (CAEMT), Western Australian School of Mines (WASM), Curtin University, Perth, WA 6102, Australia.

^2^State Key Laboratory of Intelligent Construction and Healthy Operation and Maintenance of Deep Underground Engineering, Guangdong Provincial Key Laboratory of Deep Earth Sciences and Geothermal Energy Exploitation and Utilization, College of Civil and Transportation Engineering, Shenzhen University, Shenzhen, China

*Correspondence to: zongping.shao@curtin.edu.au

**Experimental Section**

**Catalysts synthesis**

Ruthenium (III) chloride hydrate (RuCl_3_·xH_2_O, 99.98% trace metals basis) was used as the main precursor to synthesize the RuO_2_-based catalysts. Tetraethyl orthosilicate (Si(OC_2_H_5_)_4_, 98% reagent grade) and Chromium (III) chloride hexahydrate (CrCl_3_·6H_2_O, 96%) were used as silicon and chromium sources. All chemicals were purchased from Sigma–Aldrich and used as received.

The doping of Cr or Si into the Ru oxides follows a simple wet-chemistry method. Typically, 1 mmol of RuCl_3_·xH_2_O was first fully dissolved in 100 mL of ultrapure water, along with *x* mmol of CrCl_3_·6H_2_O or *y* mmol of Si(OC_2_H_5_)_4_, where x and y ranged between 0.02 and 0.1. D-Glucose (C_6_H_12_O_6_, 99.5%) and hexamethylenetetramine (C_6_H_12_N_4_, 99%) were added at a 1:1 molar ratio based on the total metal content. The mixture was stirred at room temperature for 24 hours to ensure thorough dissolution and mixing of all components. Water was then slowly evaporated at 250 ℃ to obtain a solid residue, which was placed in an alumina crucible and calcined at 600 °C in air for 5 h to finally obtain the oxide powder. The resulting powder was washed with deionized water and centrifuged for several times, then fully dried under 100 ℃ to yield either CrRuO_2_ or SiRuO_2_ catalysts. The undoped RuO_2_ (referred to as syn-RuO_2_) was synthesized using the same procedure without adding any dopants, and under the same calcination conditions.

To prepare the hierarchical Si@CrRuO_2_ oxide, a two-step synthesis route was applied. First, 100 mg of the as-obtained CrRuO_2_ powder was mixed with Si(OC_2_H_5_)_4_ (with an atomic ratio of Si: Ru = 0.08: 1) using ultrapure water as the medium and were ground in a mortar for 30 min. The resulting mixture was dried under ambient conditions and then calcined in air at 600 °C for 2 h. The calcined powder was subsequently washed, centrifuged, and dried to obtain the Si- and Cr-dual-modified catalyst. Prior to electrochemical testing and characterizations, all catalyst powders were stored in sealed glass vials.

**Material characterizations**

Transmission electron microscopy (TEM) and high-resolution TEM (HRTEM) images were acquired on FEI F200X G2 microscope. High-angle annular dark-field scanning transmission electron microscopy (HAADF-STEM) imaging and electron energy loss spectroscopy (EELS) analyses were performed on FEI, Titan Cubed Themis G201 to demonstrate the atomic-scale structure of the synthesized Si- and Cr-dual-modified RuO_2_ particle. Elemental distributions of Ru, Cr, O and Si in the catalyst particle were studied via an energy-dispersive X-ray spectroscopy (EDS) detector. X-ray diffraction (XRD) patterns were collected on a Bruker D8-Advance diffractometer equipped with a Cu *K_α_* radiation source (*K_α_* = 1.5405 Å), operated at 40 kV and 40 mA. Phase identification and crystallinity analysis were performed using Bruker EVA 6.0 software with the PDF4+ ICDD database. X-ray photoelectron spectroscopy (XPS) was carried out on a Kratos AXIS Ultra DLD instrument to analyze the surface chemical states. Ru 3*p* and O 1*s* fine spectra were collected individually. Inductively coupled plasma optical emission spectroscopy (ICP-OES, PerkinElmer Optima 8300) was used to identify the doping level of Cr into the RuO_2_ lattice. Inductively coupled plasma mass spectroscopy (ICP-MS, Agilent 7700) was used to examine the anode water circulates for any dissolved elements after the stability test. In-situ differential electrochemical mass spectrometry (DEMS) measurements were performed using a homemade system equipped with a Thermo OmniStar™ mass spectrometer. ^18^O isotope labelling treatment was applied according to established protocols, [1, 2] and gas evolution was continuously monitored during CV cycling. Electron paramagnetic resonance (EPR) measurements were performed on a Bruker EMXplus instrument.

X-ray absorption spectroscopy (XAS), including X-ray absorption near-edge structure (XANES) and extended X-ray absorption fine structure (EXAFS), was employed to investigate the local electronic and coordination structures of Ru, Cr, and Si species. The Cr *K*-edge (5.989 keV) and Si *K*-edge (1.839 keV) spectra were collected in fluorescence mode at the Tender X-ray Absorption Spectroscopy beamline TPS 32A1 of the Taiwan Photon Source (TPS). The Ru *K*-edge (22.117 keV) XAS measurements were performed at the TLS 01C1 beamline using a Si(111) double-crystal monochromator. All spectra were recorded in fluorescence mode with a Lytle detector. Energy calibration was referenced to Ru metal foils. The acquired spectra were processed using the Athena and Artemis programs within the Demeter package, following standard procedures for background subtraction, normalization, and Fourier transformation to obtain χ(k) and k^3^χ(k) functions for EXAFS analysis.

In-situ XAS measurements of the catalysts in acidic OER across various potentials and current densities were through a customized electrochemical flow cell configured a three-electrode system. Carbon paper supported catalyst layer was used as the working electrode, while Pt mesh was used as the counter electrode, together with a mercury/mercurous sulphate as the reference electrode (MSE, Hg/Hg_2_SO_4_, saturated with K_2_SO_4_, $E^{0}=+0.64 V vs. NHE$ at 25 °C). 0.5 M H_2_SO_4_ was used for creating acidic reaction environment. The potentials and current densities were applied using VersaSTAT 4 (V4, Princeton) potentiostat. During the operation, the cell was maintained at room temperature with continuous electrolyte circulation to ensure both sufficient access to the water reactant and to minimize gas bubble attachment on the catalyst layer.

**Electrochemical measurements**

The electrocatalytic OER performance of the synthesized Ru oxides was first evaluated using a rotating disk electrode (RDE, Pine MSR) connected to a CHI760E electrochemical workstation. A standard three-electrode setup was employed, with a carbon rod as the counter electrode and an Ag/AgCl (saturated KCl) as the reference electrode. The reference electrode was calibrated prior to use. A pre-polished glassy carbon RDE (geometric area: 0.196 cm^2^) was used as the working electrode. Catalyst inks were prepared by mixing 10 mg of oxide powder, 2 mg of Ketjen black, 100 μL of 5 wt.% Nafion (EW1100), 200 μL of ultrapure water, and 700 μL of ethanol. The mixture was ultrasonicated for 1 h to ensure uniform catalyst dispersion. To prepare the working electrode, 10 μL of the well-dispersed catalyst ink was drop-cast onto the electrode surface to achieve a catalyst loading of 0.5 mg cm^–2^.

The electrochemical OER performance was measured in O_2_-saturated 0.5 M H_2_SO_4_ electrolyte at room temperature. Cyclic voltammetry (CV) was performed between 0 and 1.0 V at a scan rate of 100 mV s^–1^, followed by linear sweep voltammetry (LSV) at a scan rate of 5 mV s^–1^ from 0.8 to 1.6 V vs. Ag/AgCl, and under a rotation speed of 1600 rpm. Electrochemical impedance spectroscopy (EIS) was recorded with an AC amplitude of 5 mV over a frequency range of 100 kHz to 100 mHz, and the results were averaged from three measurements for each sample. Chronoamperometry (CP) was carried out at a current density of 10 mA cm^–2^, and the potential response was monitored over time. A 95% iR compensation was applied to the LSV curves. The applied potentials were converted to the reversible hydrogen electrode (RHE) scale using the following equation:

$$E_{RHE}=E_{Ag/AgCl}+0.059 pH+0.196 V$$

The electrochemically active surface area (ECSA) of the catalysts was estimated from the electrochemical double-layer capacitance ($C_{dl}$), determined via CV scans in a non-Faradaic region (0.7–0.8 V vs. RHE) at scan rates of 10, 20, 50, 100, 200, and 250 mV s^‒1^. N_2_-saturated 0.5 M H_2_SO_4_ solution was used as the electrolyte. The ECSA was calculated based on a specific capacitance ($C_{s}$) of 0.035 mF cm^‒2^.

**PEMWE tests**

A customized PEMWE with an active area of 2 × 2 cm^2^ active area was used to evaluate the catalyst performance under practical operating conditions. The synthesized catalysts were used for the anode, while Pt/C (60 wt.% on Ketjen Black, Fuel Cell Store) served as the cathode. Nafion™ NR212 (~50 μm) was used to fabricate the catalyst-coated membranes (CCMs). For the anode catalyst ink, 40 mg of the synthesized catalyst was dispersed in Nafion dispersion (12 wt.% ionomer-to-catalyst ratio) using an isopropyl alcohol/H_2_O (2:1 v/v) mixture as the solvent. A manual spray gun (HD-131, Taiwan) was applied to coat the catalyst onto the membrane at controlled metal loadings. The coating process was performed on a hot plate maintained at 98 ℃ to ensure rapid solvent evaporation during spraying. Commercial RuO_2_ (Sigma-Aldrich) and IrO_2_ (Fuel Cell Store) were used as reference catalysts, with the CCMs prepared following the same ink formulation and coating procedure. For the cathode, the catalyst ink was prepared by mixing 20 mg of Pt/C with Nafion solution at a 60 wt.% ionomer-to-carbon ratio, followed by spray coating onto the opposite side of the membrane. The Pt loading was maintained at 1.0 mg cm^–2^ for all tested anodes to ensure consistent cathode performance. After deposition, all CCMs were dried in air at 80 °C for 1 h prior to use.

PEMWE performance testing was carried out using a single-channel power supply (Keysight, E36231A) and a potentiostat (Zahner, ZENNIUM) for galvanostatic EIS measurements. Prior to testing, ultrapure water was heated to 80 °C and circulated through both the anode and cathode to preheat the electrolyzer and maintain a stable operating temperature throughout the experiment. Electrochemical activation was performed by stepping the current sequentially to 100, 200, and 400 mA cm^–2^ for 2 min at each step to activate the electrodes. Polarization curves were obtained by stepping the voltage from 1.2 V to 1.8 V in 10 mV increments, holding each step for 1 minute to ensure stabilization before recording the current response. The PEMWE performance was evaluated both with and without iR correction. EIS measurements were conducted under galvanostatic mode at 100 mA cm^‒2^ with a 10% DC current perturbation, and the Nyquist plots were collected across a frequency range of 10 kHz to 100 mHz. The stability test was carried out by applying a constant current density of 2 A cm^‒2^ or square potential wave between 1.55 and 1.85 V to the electrolyzer while recording the voltage or current response over time.

The evaluation of the PEMWE performance for constructing the Radar plots encompasses the calculation of the energy efficiency ($\eta_{LHV}$) and specific electricity consumption (SEC). Taking the PEMWE employing Si@CrRuO_2_ anode as an example, the SEC was calculated based on operation at a current density of 2 A cm^–2^ with an applied voltage of 1.65 V for 1 hour. The normal volume of hydrogen generated per hour ($V_{H_{2}}$) under these operating conditions can be calculated as follows. Here, $n_{H_{2}}$ is the number of moles of H_2_ produced, and $V^{0}$ is the molar volume of an ideal gas at STP, taken as 22.4 L mol^–1^. $E_{input}$ stands for the electricity input for the 1-h operation.

$$V_{H_{2}}=n_{H_{2}}\times V^{0}=\frac{2 {A cm}^{-2}\times4 {cm}^{2}\times3600 s\times22.4 L/mol}{96485 {C mol}^{-1}\times2\times1000}\approx0.003343 {Nm}^{3}$$

$$SEC=\frac{E_{input}}{V_{H_{2}}}=\frac{1.65 V\times2 {A cm}^{-2}\times4 {cm}^{2}\times1 h}{1000\times0.003343 {Nm}^{3}}\approx3.948 {kWh Nm}^{-3}$$

The lower heating value (LHV) of hydrogen is 3.0 ${kWh Nm}^{-3}$; therefore, the efficiency can be calculated using the following equation:

$$\eta_{LHV}=\frac{{LHV}_{H_{2}}}{SEC}\times100\%=\frac{3.0 {kWh Nm}^{-3}}{3.948 {kWh Nm}^{-3}}\times100\%=75.9\%$$

**Density functional theory (DFT) calculations**

DFT calculations were carried out using the Vienna Ab initio Simulation Package (VASP). [3, 4] The Perdew–Burke–Ernzerhof (PBE) [5] functional within the generalized gradient approximation (GGA) was employed. Structural models of syn-RuO_2_, CrRuO_2_, and Si@CrRuO_2_ were constructed based on XRD profiles of the synthesized catalyst powders. For the RuO_2_ (101) surface, a 2 × 2 supercell with dimensions of 10.91 Å × 8.97 Å was adopted, derived from lattice parameters of 5.46 Å × 4.49 Å. Brillouin zone sampling was performed using a Monkhorst–Pack *k*-point mesh of 2 × 3 × 1. The projected augmented wave (PAW) method was used to describe ionic cores, with valence electrons expanded in a plane-wave basis set using a kinetic energy cutoff of 450 eV. [6, 7] Geometry optimizations were conducted until the residual forces on all unconstrained atoms were below 0.02 eV Å^–1^. Van der Waals interactions were accounted for using the DFT-D3 empirical correction scheme. In all calculations, atoms in the bottom half of the slab were fixed to mimic bulk constraints. The calculation of the oxygen vacancy formation energy (${\Delta E}_{O_{v}}$) follows the equation below, where $E_{O_{2}}$ is energy of oxygen, $E_{O}$ stands for the surface energy before oxygen loss.

$${\Delta E}_{O_{v}}=E_{O_{v}}+\frac{1}{2}E_{O_{2}}-E_{O}$$

**Supplementary Figures and Tables**


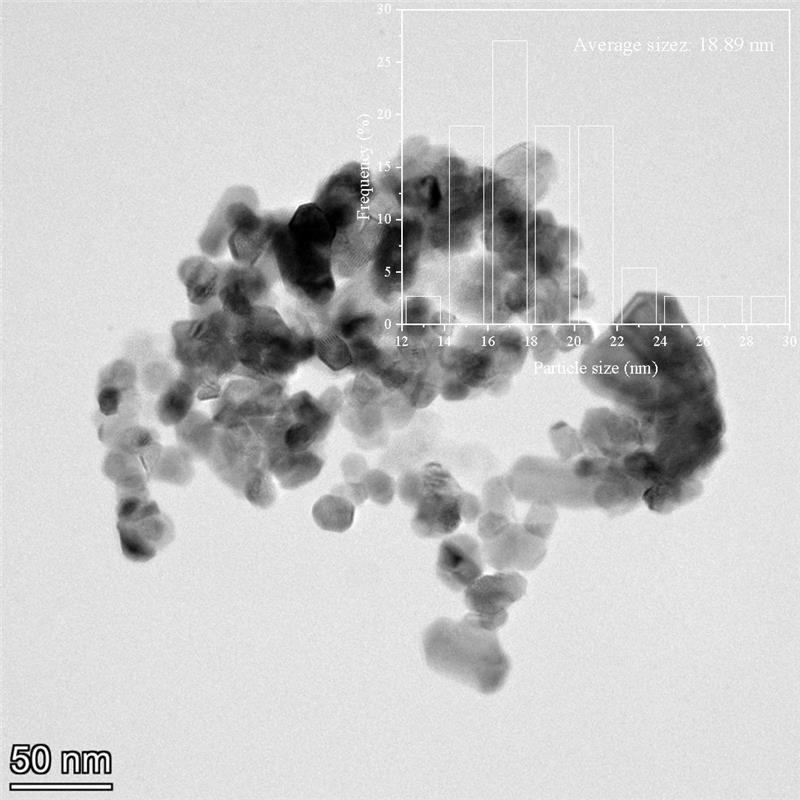


**Figure S1**. TEM image and average particle size analysis of the Cr doped CrRuO_2_.


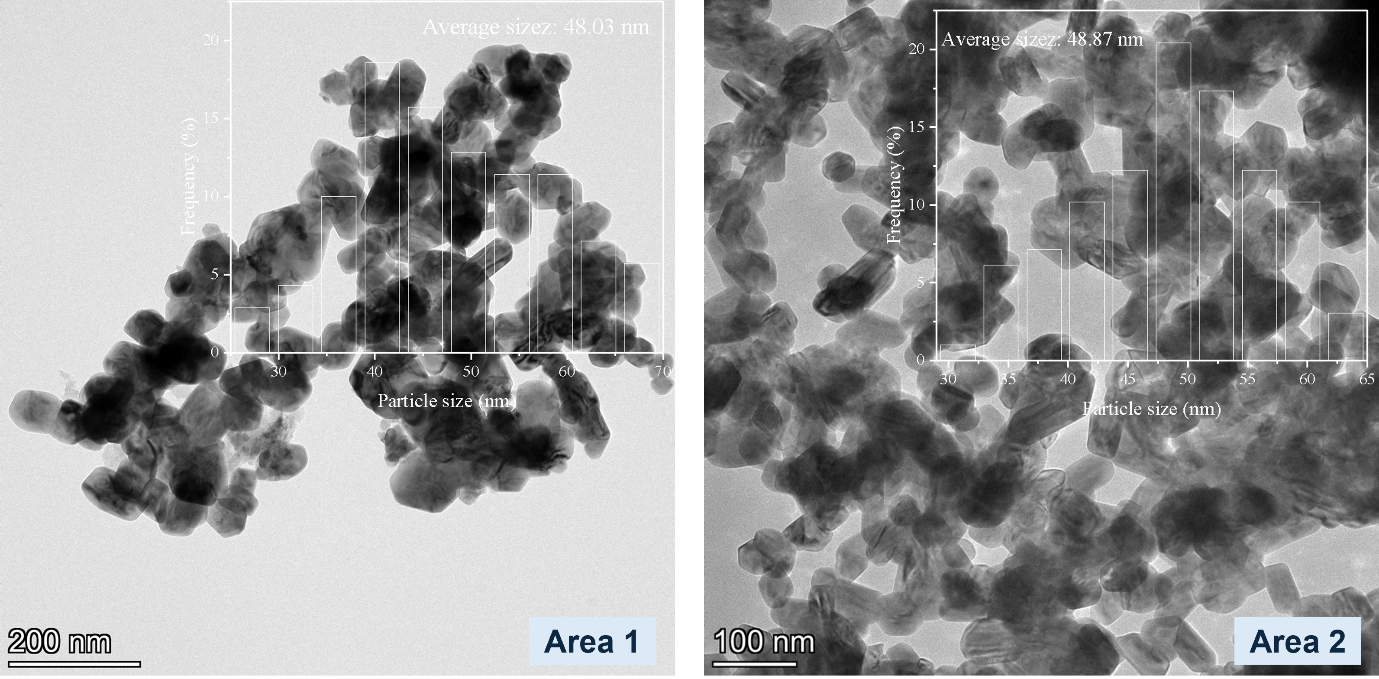


**Figure S2**. TEM images and average particle size analysis of the syn-RuO_2_.


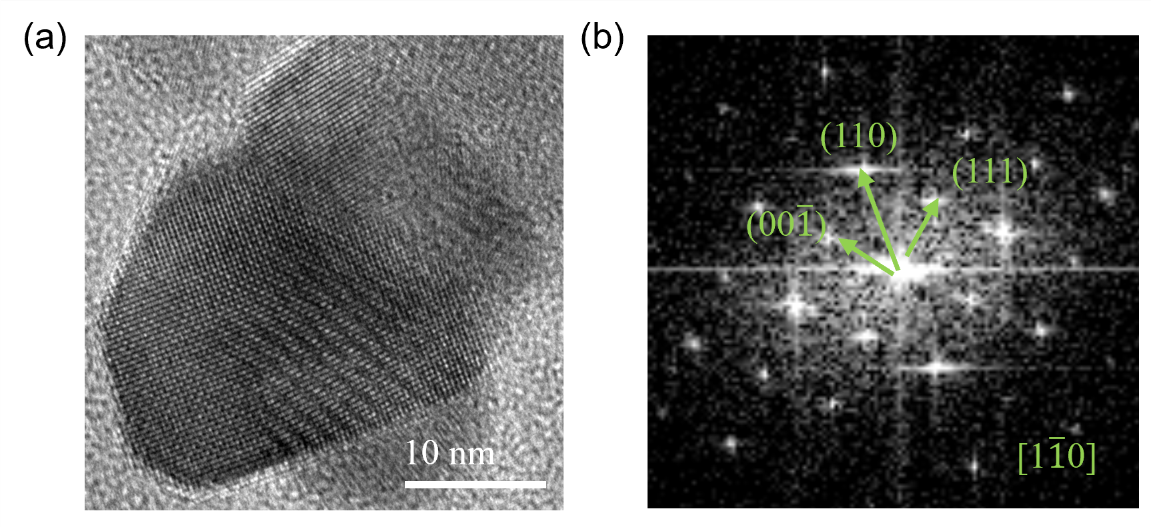


**Figure S3**. (a) HR-TEM image of a random CrRuO_2_ particle and (b) the fast Fourier transform

diffraction pattern of the area.


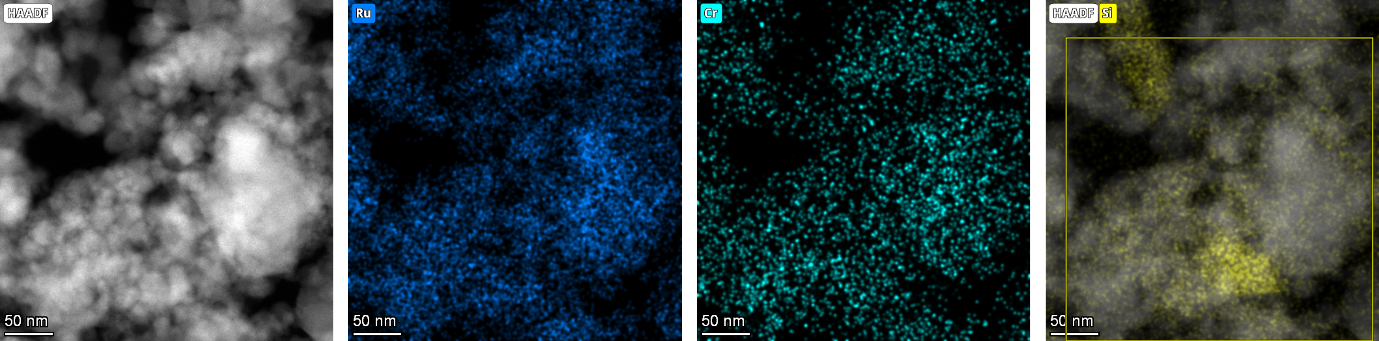


**Figure S4**. A broader view of the synthesized Si@CrRuO_2_ particles. HAADF-STEM image of a selected region and corresponding EDS elemental mapping showing the distribution of Cr in the Ru oxide lattice, and the surface modification of Si. Scale bar: 50 nm.

**
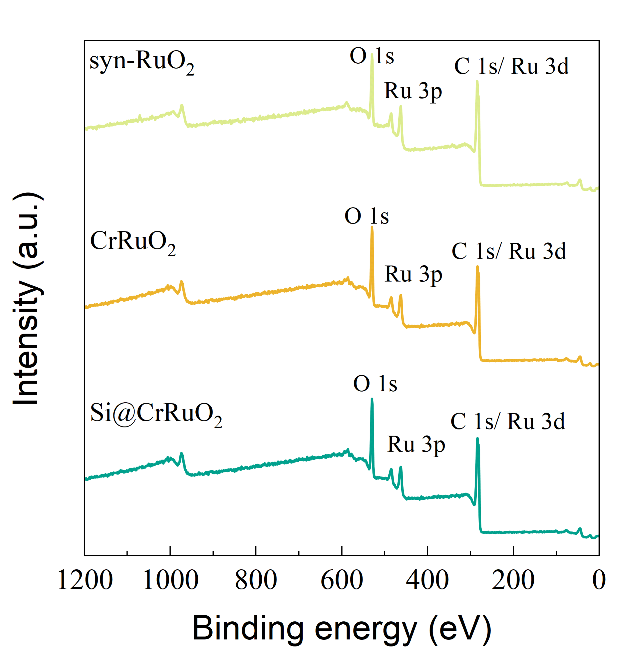
**

**Figure S5**. XPS survey spectra of the synthesized Ru oxides with characteristic O 1*s*, Ru 3*p*, C 1*s*, and Ru 3*d* orbital features.

**
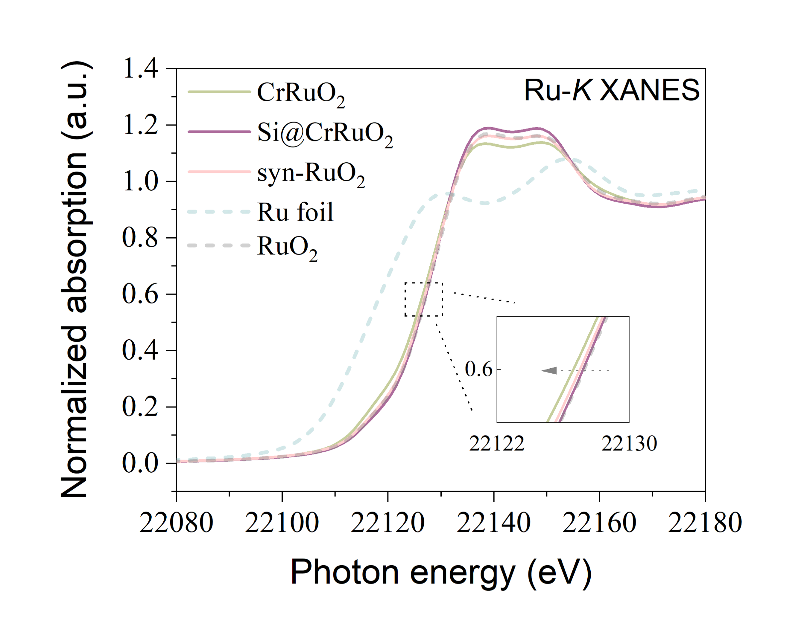
**

**Figure S6.** Ru *K*-edge XANES spectra of the synthesized Ru oxides powder. Reference spectra of Ru foil and RuO_2_ were used for edge calibration and valence comparison.


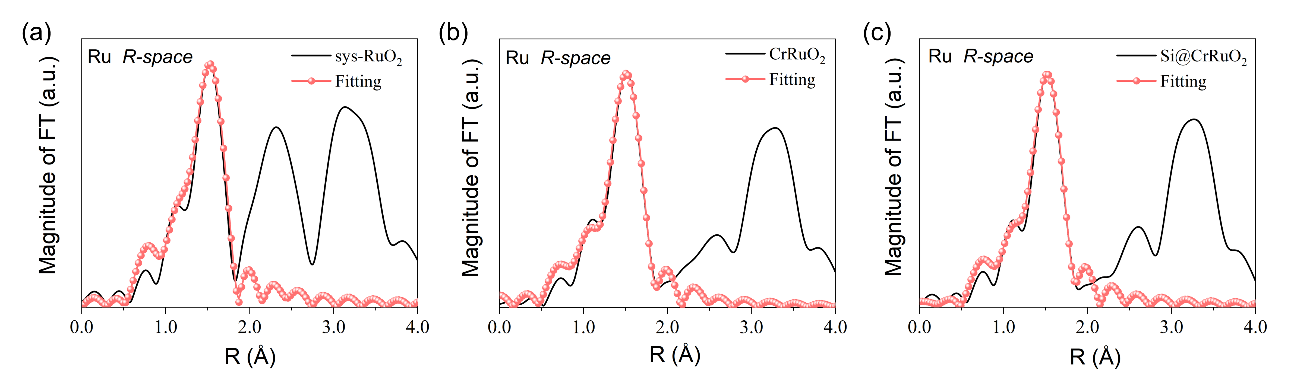


**Figure S7**. *R*-space fitting of the FT-EXAFS spectra of the synthesized Ru oxide powders.


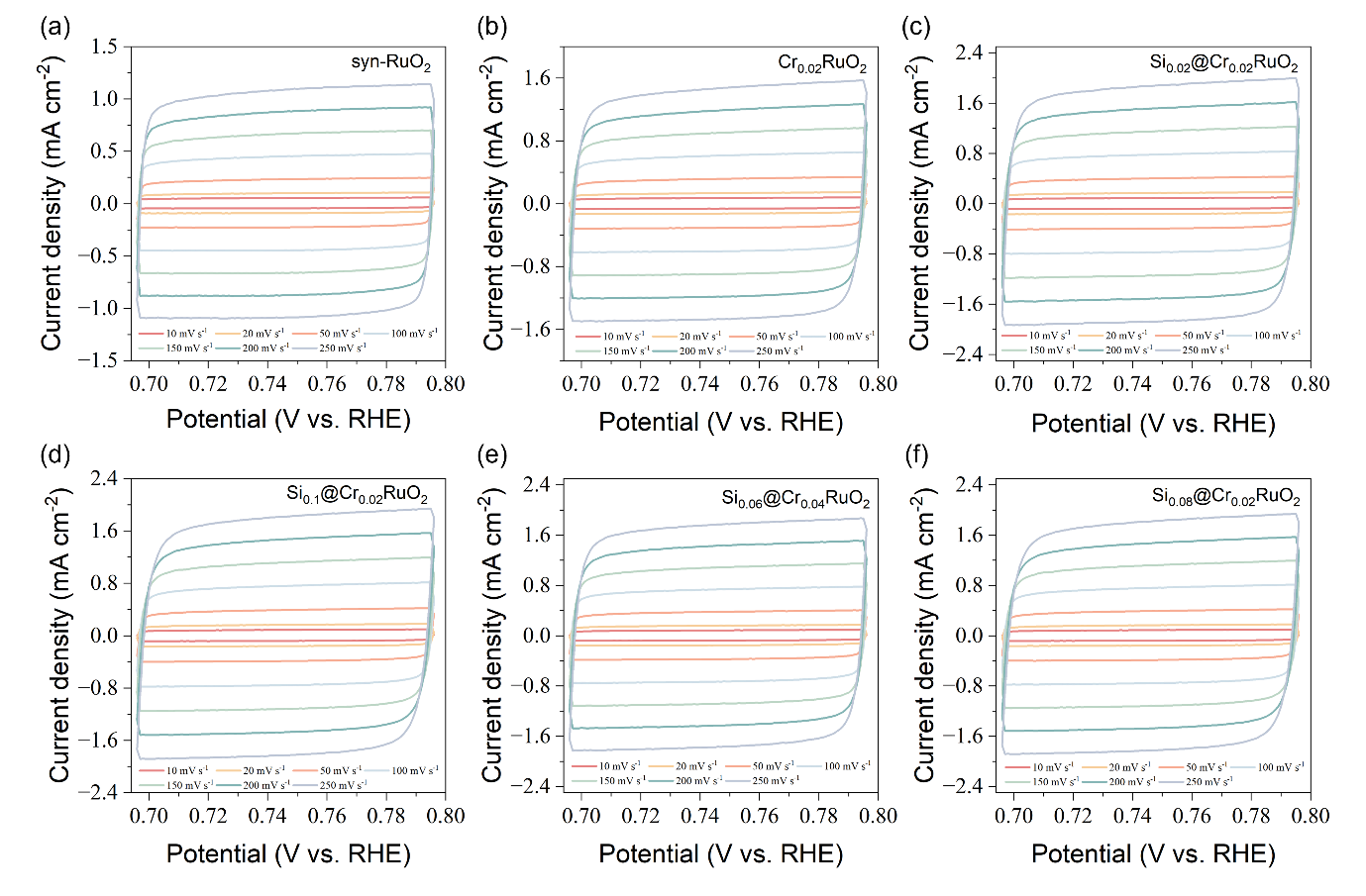


**Figure S8**. CV scans in a non-Faradaic region (0.7–0.8 V vs. RHE) at scan rates of 10, 20, 50, 100, 200, and 250 mV s^‒1^ for the synthesized catalysts: (a) syn-RuO_2_, (b) Cr_0.02_RuO_2_, (c) Si_0.02_@Cr_0.02_RuO_2_, (d) Si_0.1_@Cr_0.02_RuO_2_, (e) Si_0.06_@Cr_0.04_RuO_2_, and (f) Si_0.08_@Cr_0.02_RuO_2_.


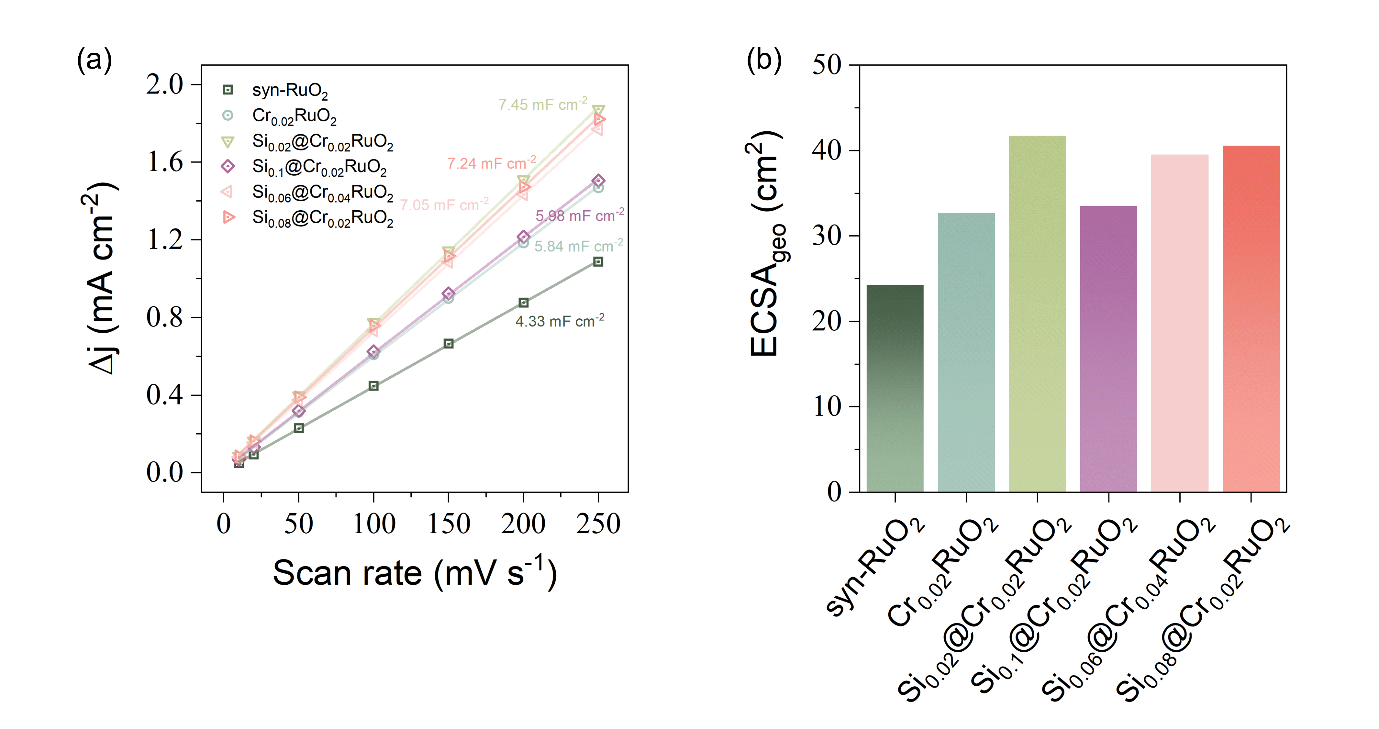


**Figure S9**. (a) Capacitive current density at 0.746 V (vs. RHE) as a function of scan rates and the corresponding $C_{dl}$ values. (b) The calculated geometric ECSAs for the synthesized catalysts.

**
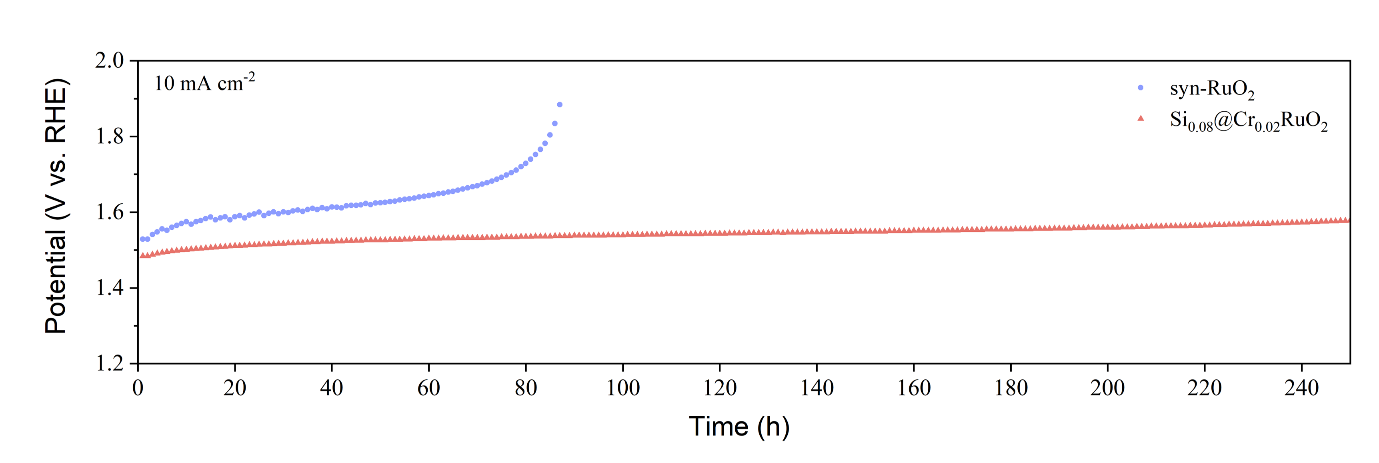
**

**Figure S10.** Chronopotentiometric stability test of the Si_0.08_@Cr_0.02_RuO_2_ compared with syn-RuO_2_ under RDE conditions at 10 mA cm⁻^2^ and room temperature.


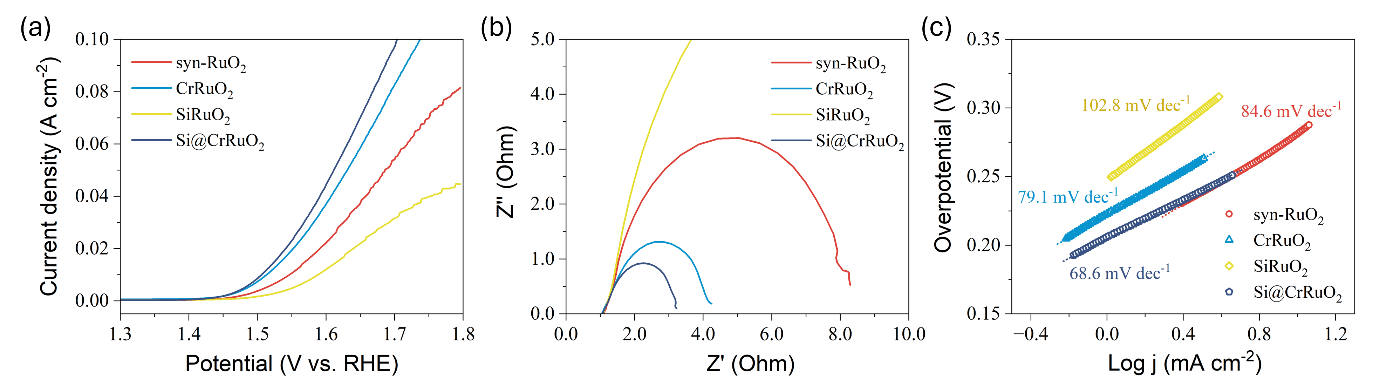


**Figure S11**. (a) Geometry-normalized LSV curves obtained in 0.5 M H_2_SO_4_ electrolyte comparing the synthesized Ru oxides. (b) Nyquist plots from EIS measurements. (c) Tafel plots derived from steady-state polarization curves in the activation zone.

**
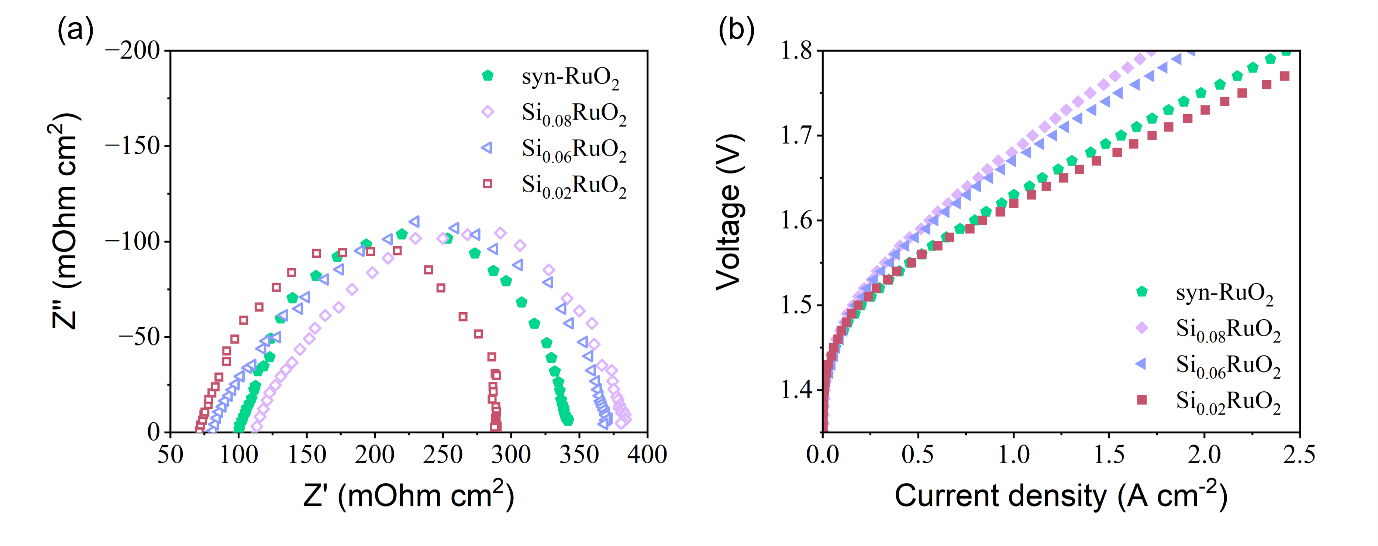
**

**Figure S12.** (a) Nyquist plots from galvanostatic EIS measurements of PEMWE operating at 100 mA cm^–2^. (b) PEMWE performance curves using Si-doped RuO_2_ anodes with varying Si doping levels.


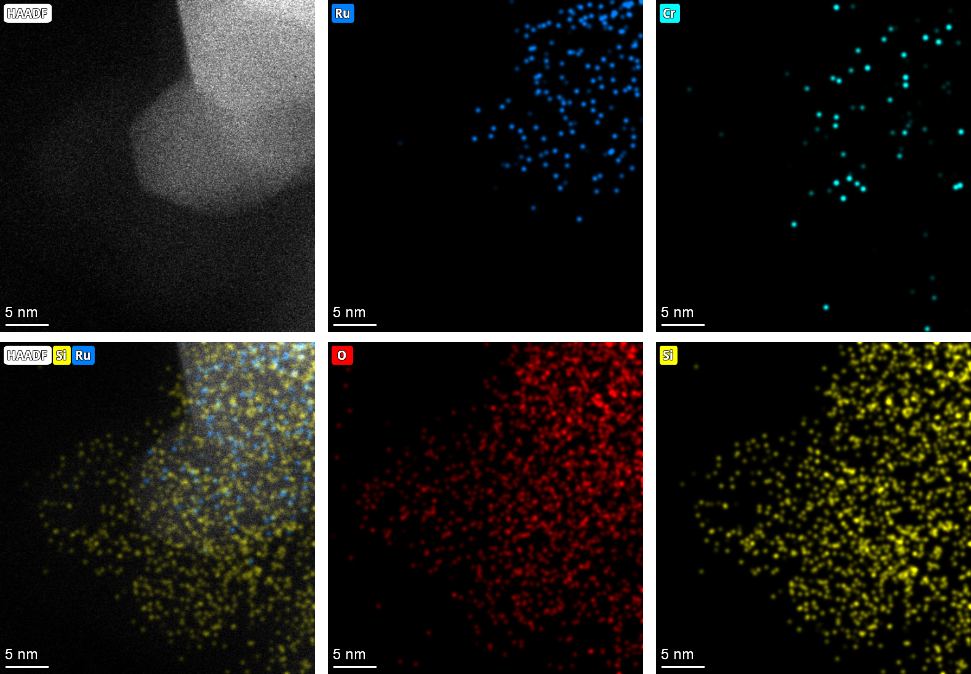


**Figure S13**. Morphology of Si@CrRuO_2_ with increasing Si modification level to 10 at.%. HAADF-STEM image of a selected particle and corresponding EDS elemental mapping showing the distribution of Ru, O, Cr, and Si. Scale bar: 5 nm.

**
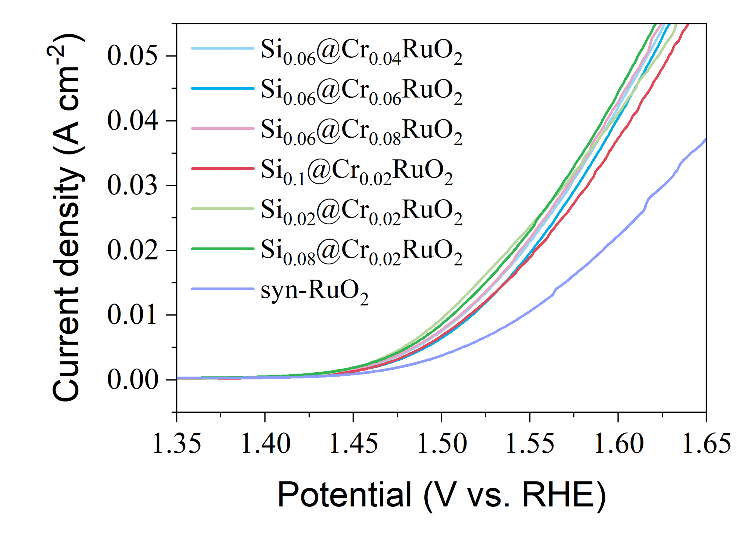
**

**Figure S14.** LSV curves of a series of Si@CrRuO_2_ catalysts with varying Si and Cr modification levels.

**
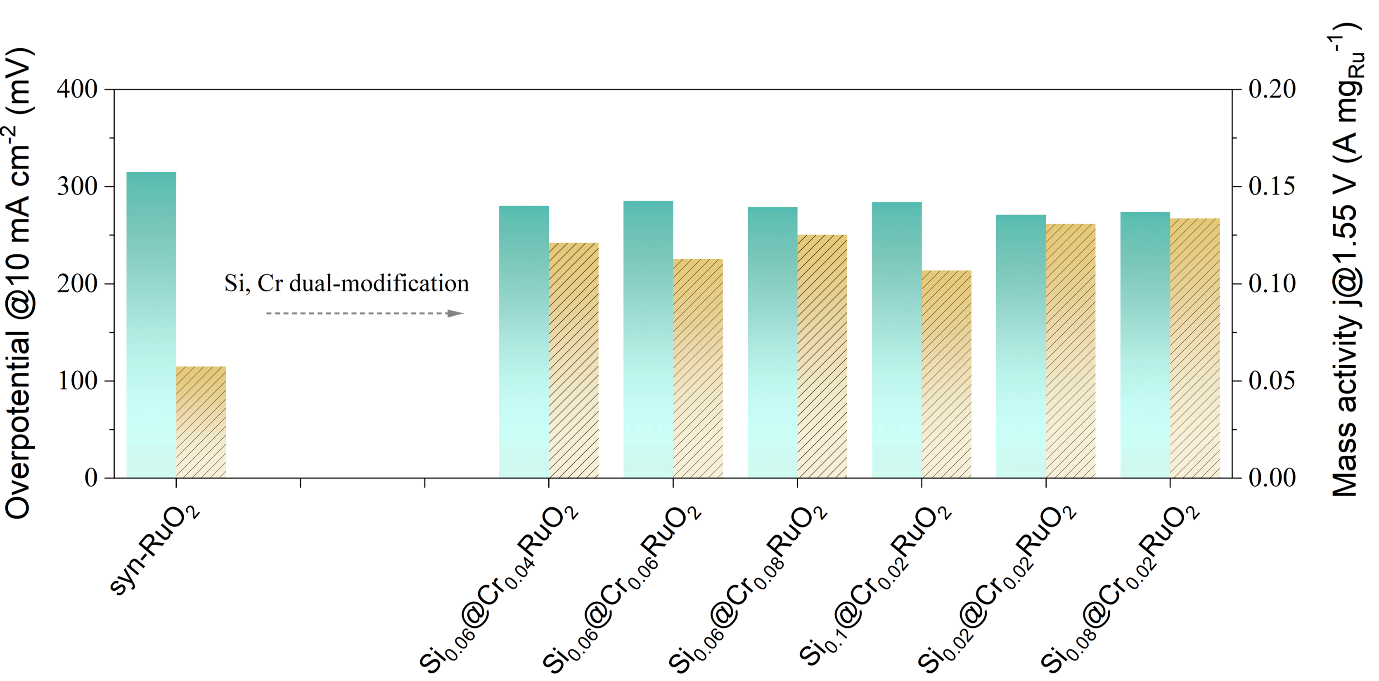
**

**Figure S15.** Bar plot comparison of the OER overpotentials and mass activities with varying Si and Cr modification levels.


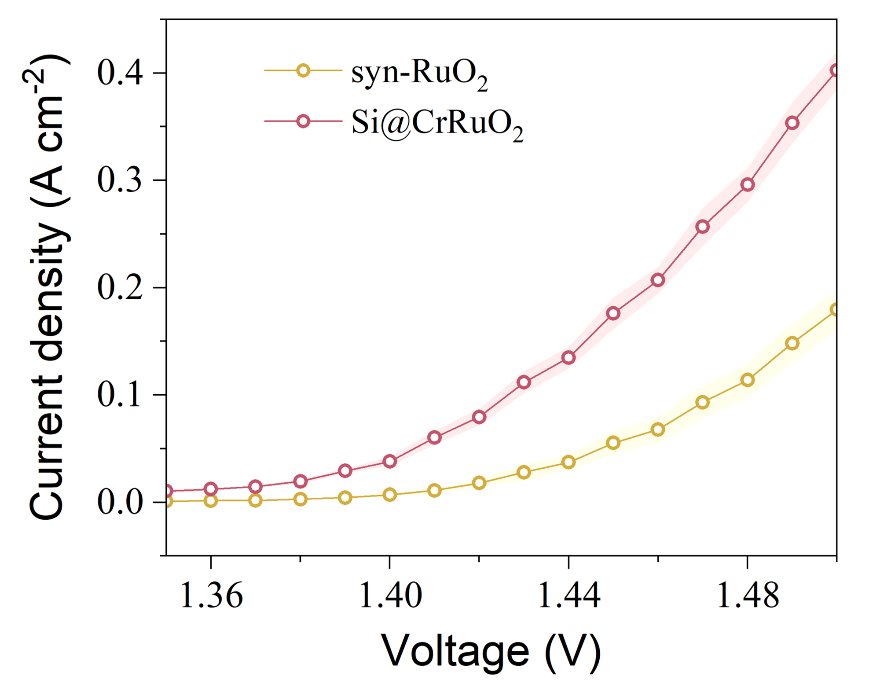


**Figure S16.** PEMWE performance curves with error bars showing consistency across three independent measurements.


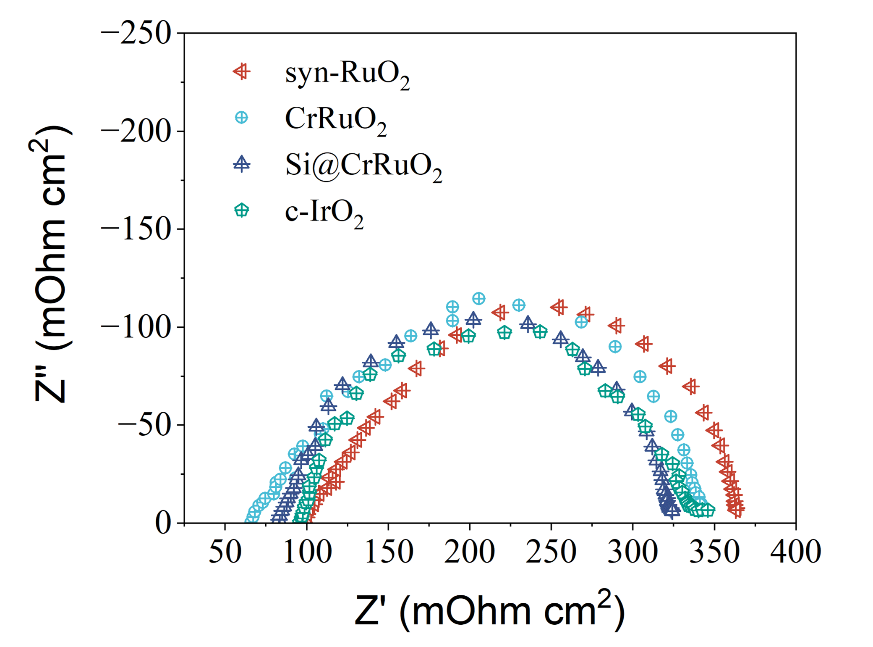


**Figure S17**. Original galvanostatic EIS spectra used for DRT fitting and analysis, obtained from PEMWEs operating at 100 mA cm^–2^ with synthesized Ru-oxide catalysts as the anode, and the commercial IrO_2_ catalyst for comparison. High-frequency resistance (HFR) values are summarized in Table S3.


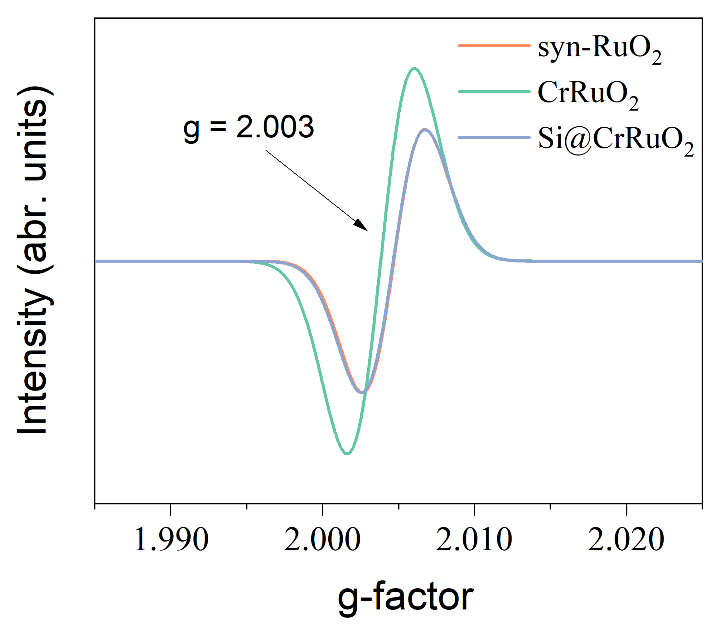


**Figure S18**. EPR spectra of the synthesized Ru oxide catalysts. The marked resonance signal at g = 2.003 corresponds to oxygen vacancy defects.


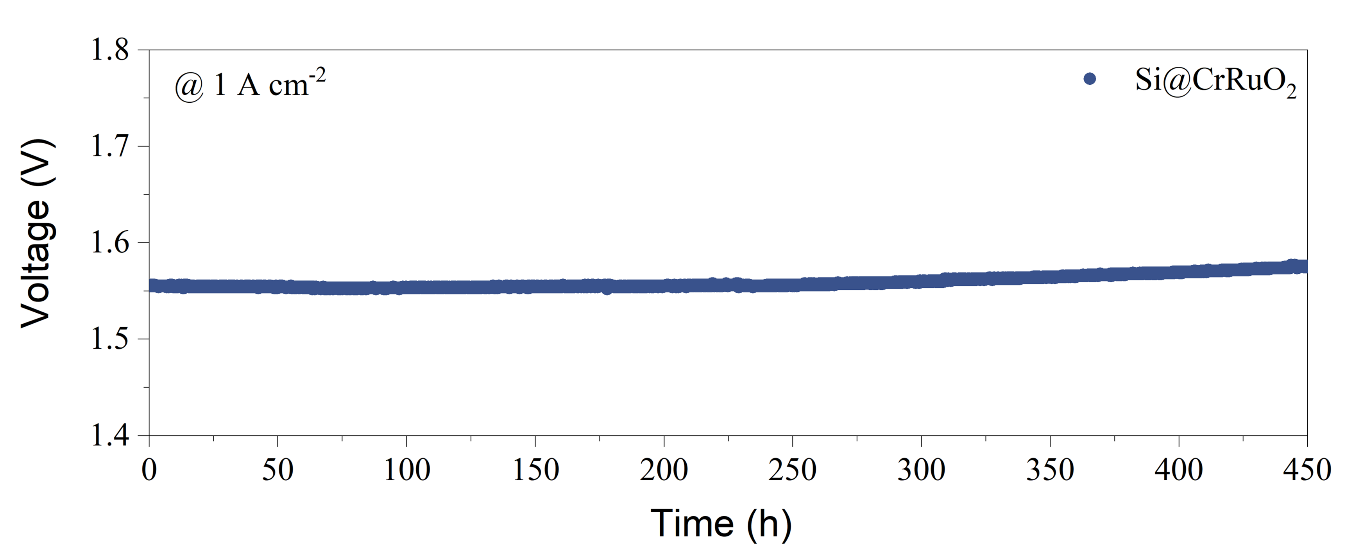


**Figure S19**. Stability test of the PEMWE with Si@CrRuO_2_ anode at 1 A cm⁻^2^. PEMWE with an active area of 2 × 2 cm^2^ was operated at 80 °C with pure water supplied to both the cathode and anode.


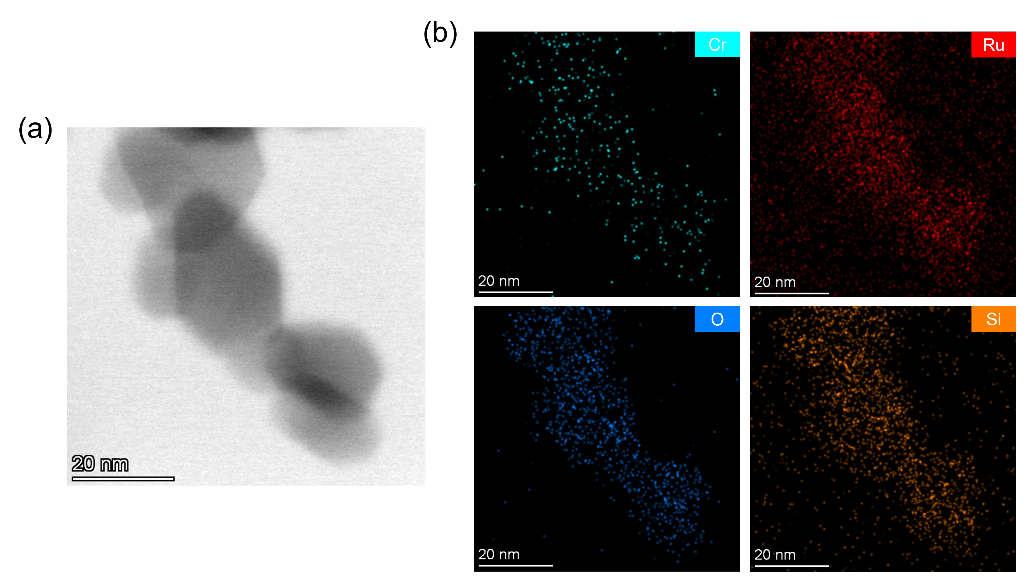


**Figure S20**. (a) TEM image of the Si@CrRuO_2_ catalyst particles after the stability test conducted at 2 A cm^–2^ for 10 h, and (b) the EDS elemental mapping showing the distribution of Ru, Cr, O and Si.


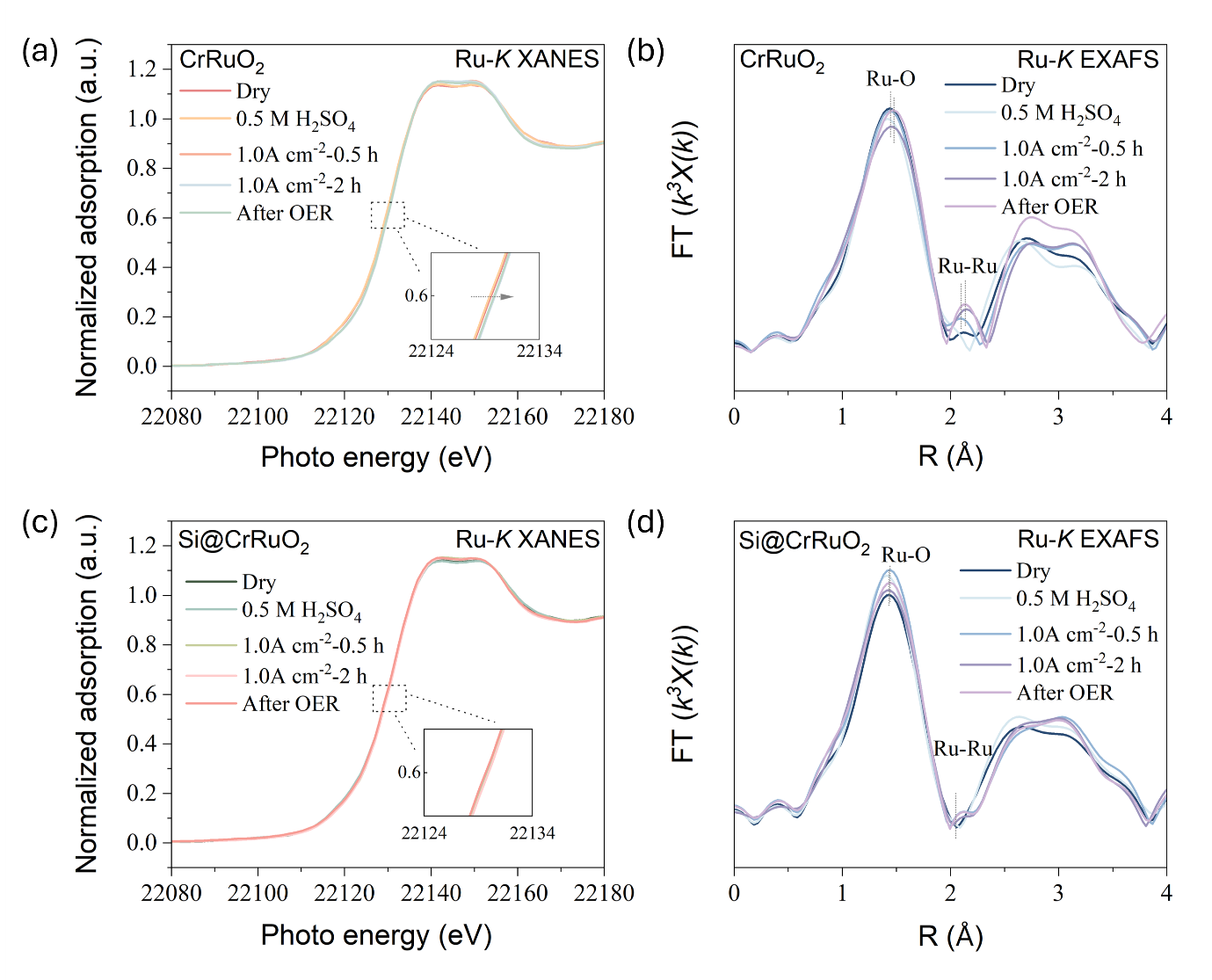


**Figure S21**. In-situ (a) Ru *K*-edge XANES spectra, and (b) Ru *K*-edge EXAFS spectra of the CrRuO_2_ catalyst prior and after the OER reaction under 1.0 A cm^–2^. In-situ (c) Ru *K*-edge XANES spectra, and (d) Ru *K*-edge EXAFS spectra of the Si@CrRuO_2_ catalyst prior and after the OER reaction.


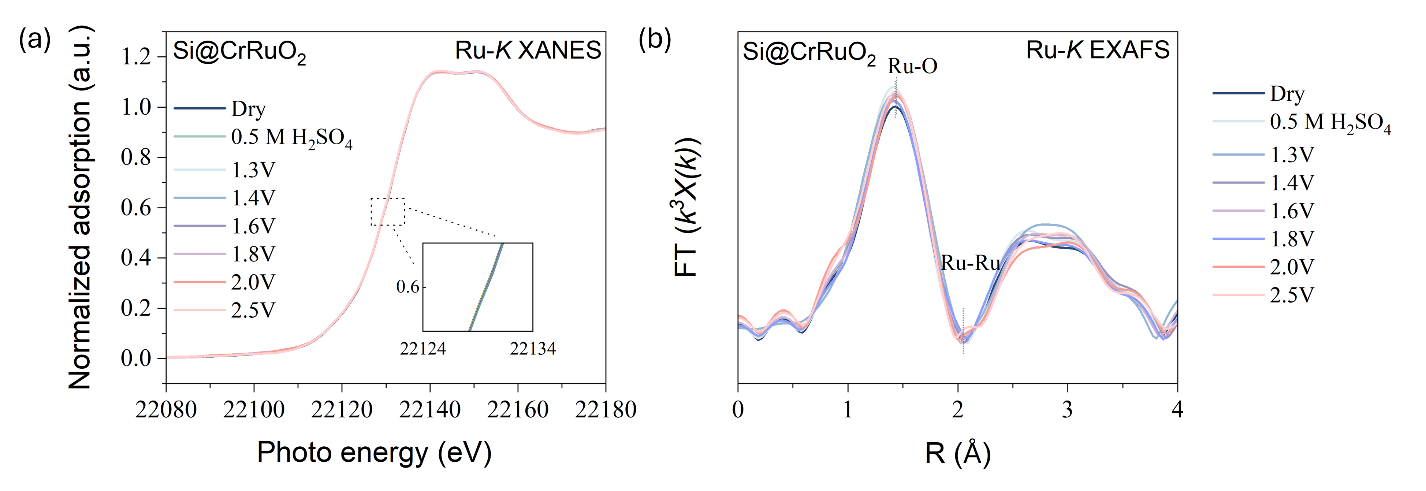


**Figure S22**. In-situ (a) Ru *K*-edge XANES spectra, and (b) Ru *K*-edge EXAFS spectra of the Si@CrRuO_2_ catalyst prior and after the OER reaction under different voltages.


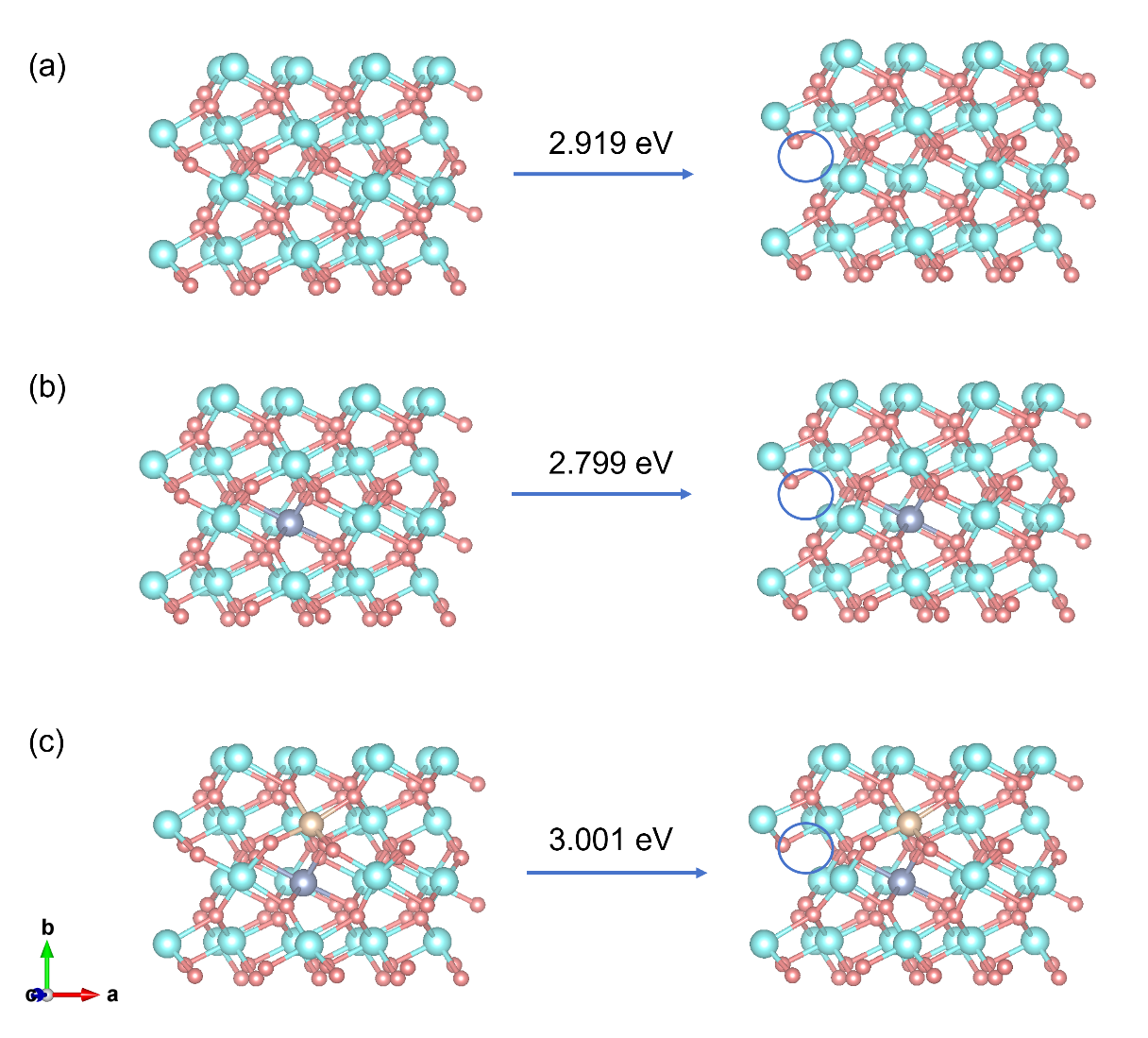


**Figure S23**. (a) Optimised structure of syn-RuO_2_ (101) with perfect surface and structural evolution with O_v_ formation energy. (b) Optimised structure of CrRuO_2_ and structural evolution with O_v_ formation energy, and (c) Optimised structure of Si@CrRuO_2_ and structural evolution with O_v_ formation energy. The blue, pink, purple, and gold balls represent Ru, O, Cr, and Si atoms respectively. The O_v_ has been circled in the picture.


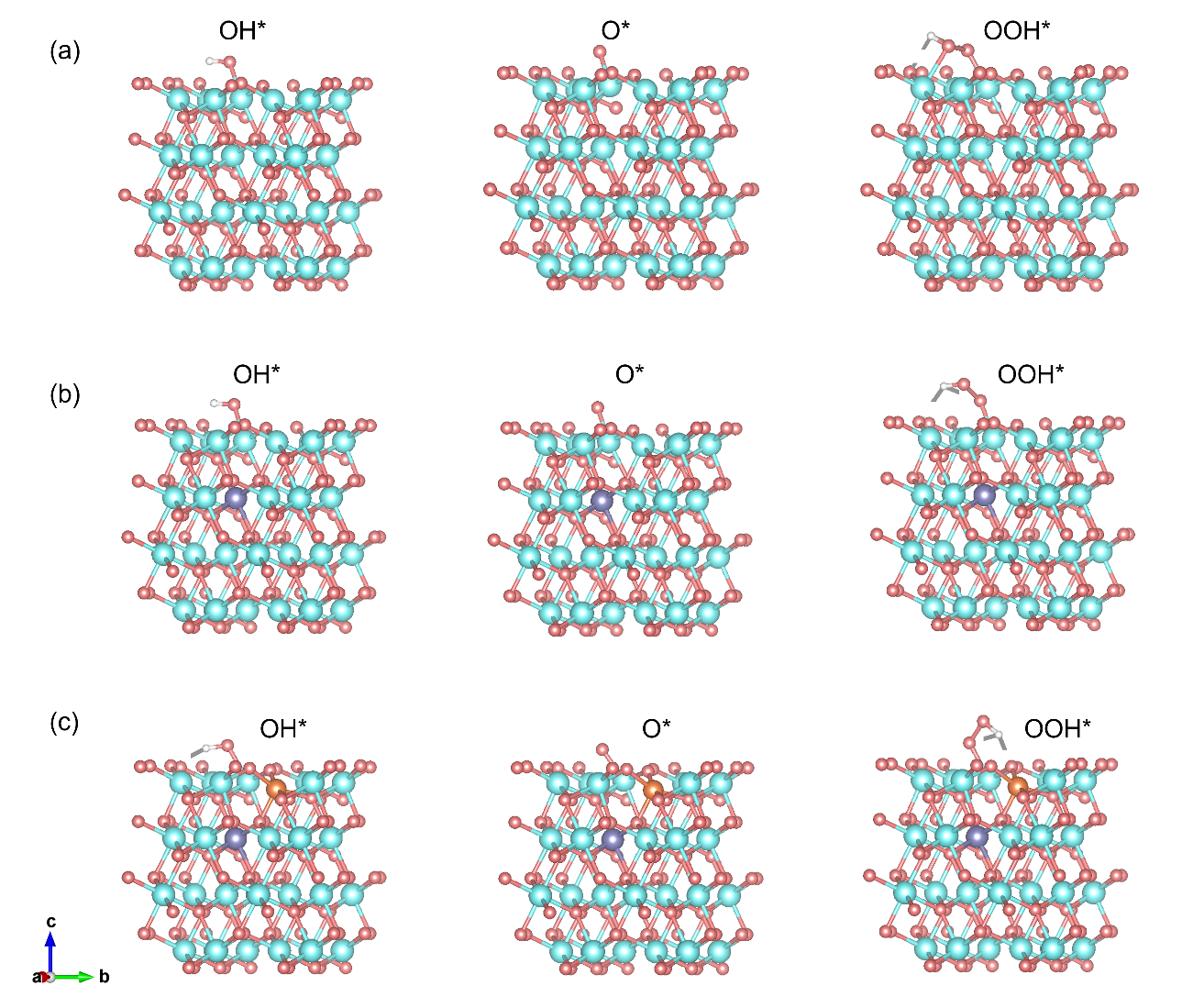


**Figure S24**. The intermediates adsorption during the OER pathway on (a) the optimised syn-RuO_2_-O_v_ (101) surface, (b) the optimised CrRuO_2_-O_v_ surface, and (c) the optimised Si@CrRuO_2_-O_v_ surface. The blue, pink, purple, gold and white balls represent Ru, O, Cr, Si and H atoms respectively.


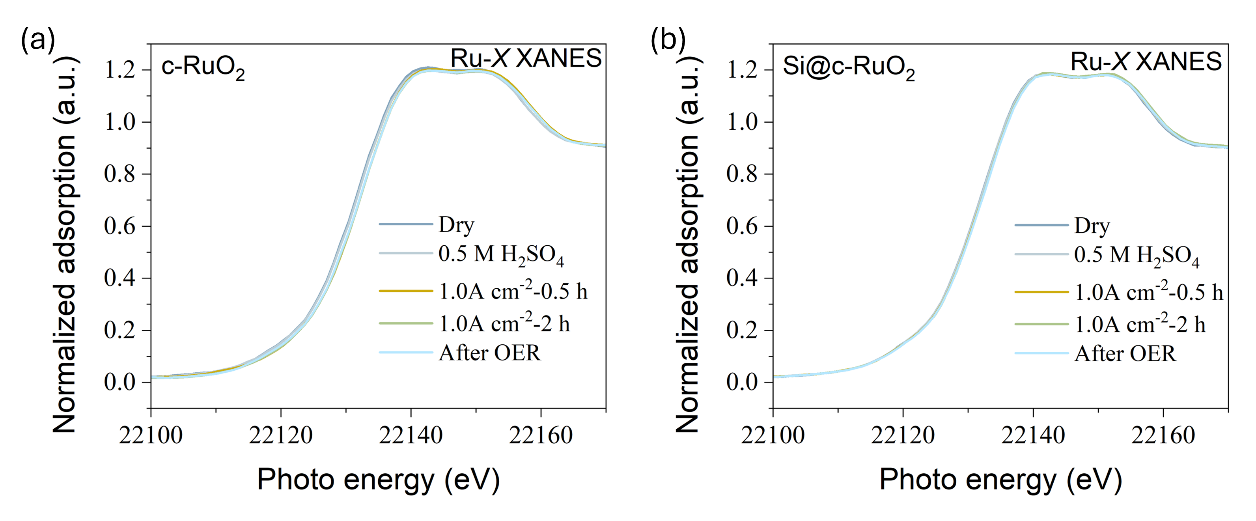


**Figure S25**. In-situ Ru *K*-edge XANES spectra of the (a) c-RuO_2_ catalyst and (b) Si@c-RuO_2_ catalyst prior and after the OER reaction under 1.0 A cm^–2^.


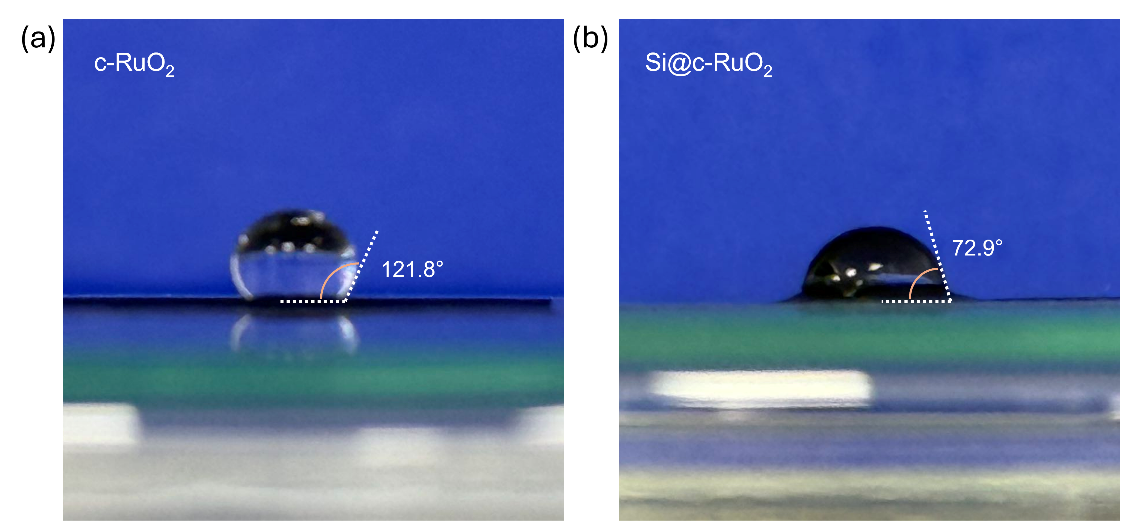


**Figure S26**. (a) A hydrophobic c-RuO_2_ catalyst layer with a measured water contact angel of approx. 121.8°. (b) A relatively hydrophilic Si@c-RuO_2_ catalyst layer with a water contact angel of approx. 72.9°.


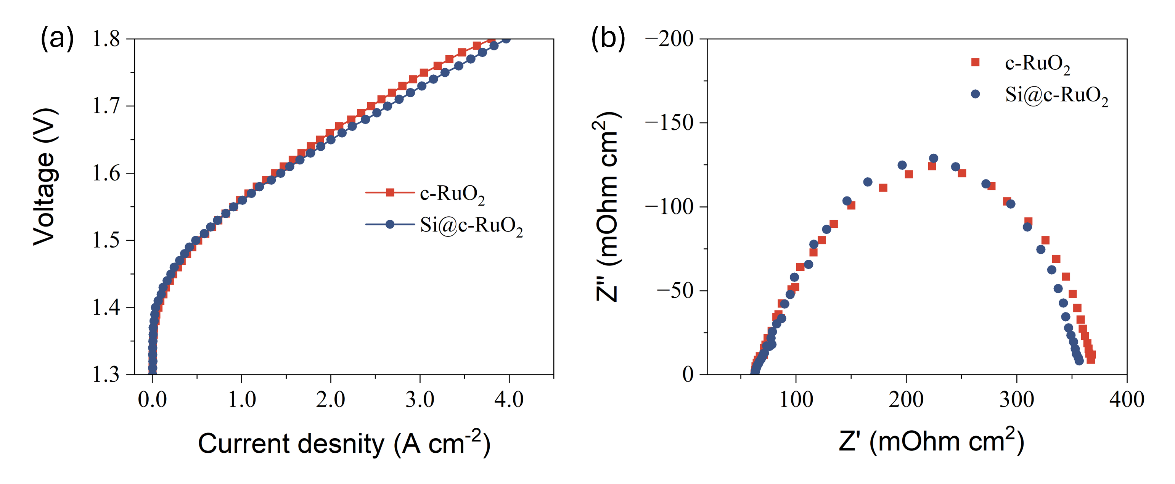


**Figure S27**. (a) PEMWE performance curves using c-RuO_2_ anode and Si-doped Si@c-RuO_2_ anode. (b) Nyquist plots from galvanostatic EIS measurements of PEMWEs operating at 100 mA cm^–2^.

**Table S1**. ICP-OES analysis of the actual Cr doping levels in the synthesized oxides.

| **Sample** | **Cr: Ru atomic ratio** |
| --- | --- |
| Cr_0.02_RuO_2_ | 0.019 |
| Si_0.02_Cr_0.02_RuO_2_ | 0.017 |
| Si_0.08_Cr_0.02_RuO_2_ | 0.022 |
| Si_0.06_@Cr_0.08_RuO_2_ | 0.074 |
| Si_0.06_@Cr_0.04_RuO_2_ | 0.040 |

**Table S2**. Fitting parameters obtained from the FT-EXAFS analysis of the synthesized Ru oxide powders.

| Catalyst | Path | *R*-factor | Coordination numbers  (Å) | E_0_  (eV) | σ^2^  (×10⁻³ Å^2^) | Bond length (Å) |
| --- | --- | --- | --- | --- | --- | --- |
| syn-RuO_2_ | Ru–O | 0.017834 | 5.993 | 0.817 | 0.00610 | 1.9998 |
| CrRuO_2_ | Ru–O | 0.010292 | 5.713 | 0.573 | 0.00391 | 1.9904 |
| Si@CrRuO_2_ | Ru–O | 0.021684 | 5.162 | 0.380 | 0.00376 | 1.9897 |

**Table S3**. HFR values from galvanostatic EIS measurements of PEMWEs operating at 100 mA cm^–2^ using synthesized Ru-oxide anodes, and the commercial IrO_2_ anode.

| **Anode** | **HFR**  **(mOhm cm^2^)** |
| --- | --- |
| syn-RuO_2_ | 94.00 |
| CrRuO_2_ | 60.16 |
| Si@CrRuO_2_ | 76.73 |
| c-IrO_2_ | 93.37 |

**Table S4**. A comparison of the reported PEMWE stability with Ru oxide-based anodes under high-current-density operation at 1 A cm^–2^ and above.

| **Catalysts** | **PEMWE conditions** | **Current density**  **(A cm^–2^)** | **Voltage**  **(V)** | **Time (h) with decay rate**  **(if applicable)** |
| --- | --- | --- | --- | --- |
| [8]m-RuO_2_ | Nafion 115  Ru loading of 1.5 mg cm^–2^  Pure water, 60 ℃ | 1 | ~1.75 | 100 h with slow decay |
| [9]anh-RuO_2_ | Nafion 212  Ru loading of 1.0 mg cm^–2^  Pure water, 80 ℃ | 1 | 1.67 | 550 h with a decay rate of ~133 µV h^‒1^ |
| [10]SnO_2_/Nb_2_O_5_@RuO_2_ | Ru loading of 0.1 mg cm^–2^  0.5 M H_2_SO_4_ | 1 | 1.75 | 600 h without obvious decay |
| [11]La-RuO_2_@titanium mesh | Pure water, 60 ℃ | 1 | 1.815 | 120 h without any decay |
| [12](Ru–W)O_x_ | Nafion 117  (Ru–W)O_x_ loading of 2.0 mg cm^–2^  Pure water, 80 ℃ | 1 | 1.62 | 50 h |
| [13]SnRuO_x_ | Nafion 212, Nafion 115  Ru loading of 1.46 mg cm^–2^  Distilled water, 50 ℃ | 1 | 1.565 | 1300 h with a decay rate of 53 µV h^‒1^ |
| [14]Ba_0.3_(SO_4_)_δ_W_0.2_Ru_0.5_O_2−δ_ | Nafion 115  Oxide loading of ~ 3 mg cm^–2^  Distilled water, 80 ℃ | 1 | 1.68 | 300 h with the voltage increased by ~ 4.9 % (~ 274 µV h^‒1^) |
| [15]H–Mn_x_Ru_1–x_O_2_ | Nafion 115  Ru loading of 2 mg cm^–2^  Deionized water, 80 ℃ | 1 | 1.65 | 80 h with a decay rate of 450 µV h^‒1^ |
| [16]Co-Ru@RuO_2_ | Nafion 115  Ru loading of 0.34 mg cm^–2^  Deionized water, 60 ℃ | 1 | 1.58 | Obvious decay within 75 h |
| [17]ZrO_2_–RuO_2_ | Nafion 117  ZrO_2_–RuO_2_ loading of 2.5 mg cm^–2^  Pure water, 80 ℃ | 2 | 1.75 | 100 h with a decay rate of 500 µV h^‒1^ |
| [18]Pb-RuO_2_ | Nafion 212  Deionized water | 1 | 1.688 | 100 h with a decay rate of 87 µV h^‒1^ |
| [19]Cr_0.2_Ru_0.8_O_2-x_ | Nafion 115  Anode catalyst loading of 3.0 mg cm^–2^  Deionized water, 60 ℃ | 1 | 1.77 | 200 h with a decay rate of 320 µV h^‒1^ |
| [20]Ta_0.2_Ru_0.8_O_2−x_ | Nafion 115, 625 cm^2^  Anode catalyst loading of 0.8 mg cm^–2^  Deionized water, 50 ℃ | 1 | 1.704 | 2800 h at a decay rate < ~14 µV h^‒1^ |
| [21]M-RuIrFeCoNiO_2_ | Nafion 212  Noble metal loading of 2 mg cm^–2^  Deionized water, 80 ℃ | 1 | ~1.85 | 500 h (No decay rate specified) |
| [22]Er-RuO_x_ | Nafion 117  Distilled water, 80 ℃ | 1 | 1.59 | 100 h with a decay rate of ~576 µV h^‒1^ |
| **This work**: Si@CrRuO_2_ | Nafion 212  Anode catalyst loading 1.0 mg cm^–2^  Deionized water, 80 ℃ | 2 | 1.65 | 200 h with a decay rate of 64.4 µV h^‒1^ |

**Table S5**. ICP-MS analysis of dissolved elements in the anolyte after the stability test at 2 A cm^–2^ for 10 h.

| Anolyte | Ru concentration  (ppb h^–1^) | Cr concentration  (ppb h^–1^) | Si concentration  (ppb h^–1^) |
| --- | --- | --- | --- |
| syn-RuO_2_ | 11.611 | / | / |
| CrRuO_2_ | 3.544 | 0.034 | / |
| Si@CrRuO_2_ | 1.227 | 0.022 | 0 |

**Supplementary References**

[1] Y. Wen, P. Chen, L. Wang, S. Li, Z. Wang, J. Abed, X. Mao, Y. Min, C.T. Dinh, P.D. Luna, R. Huang, L. Zhang, L. Wang, L. Wang, R.J. Nielsen, H. Li, T. Zhuang, C. Ke, O. Voznyy, Y. Hu, Y. Li, W.A. Goddard Iii, B. Zhang, H. Peng, E.H. Sargent, Stabilizing Highly Active Ru Sites by Suppressing Lattice Oxygen Participation in Acidic Water Oxidation, Journal of the American Chemical Society, 143 (2021) 6482-6490.

[2] A. Grimaud, O. Diaz-Morales, B. Han, W.T. Hong, Y.-L. Lee, L. Giordano, K.A. Stoerzinger, M.T.M. Koper, Y. Shao-Horn, Activating Lattice Oxygen Redox Reactions in Metal Oxides to Catalyse Oxygen Evolution, Nature Chemistry, 9 (2017) 457-465.

[3] G. Kresse, J. Furthmüller, Efficiency of ab-initio total energy calculations for metals and semiconductors using a plane-wave basis set, Comput. Mater. Sci., 6 (1996) 15-50.

[4] G. Kresse, J. Hafner, Ab initio molecular-dynamics simulation of the liquid-metal–amorphous-semiconductor transition in germanium, Phys. Rev. B, 49 (1994) 14251-14269.

[5] J.P. Perdew, K. Burke, M. Ernzerhof, Generalized Gradient Approximation Made Simple, Phys. Rev. Lett., 77 (1996) 3865-3868.

[6] P.E. Blöchl, O. Jepsen, O.K. Andersen, Improved tetrahedron method for Brillouin-zone integrations, Phys. Rev. B, 49 (1994) 16223-16233.

[7] G. Kresse, D. Joubert, From ultrasoft pseudopotentials to the projector augmented-wave method, Phys. Rev. B, 59 (1999) 1758-1775.

[8] G. Zhao, W. Guo, M. Shan, Y. Fang, G. Wang, M. Gao, Y. Liu, H. Pan, W. Sun, Metallic Ru–Ru Interaction in Ruthenium Oxide Enabling Durable Proton Exchange Membrane Water Electrolysis, Advanced Materials, 36 (2024) 2404213.

[9] J. Tang, D. Guan, H. Xu, L. Zhao, U. Arshad, Z. Fang, T. Zhu, M. Kim, C.-W. Pao, Z. Hu, J. Ge, Z. Shao, Undoped Ruthenium Oxide as a Stable Catalyst for the Acidic Oxygen Evolution Reaction, Nature Communications, 16 (2025) 801.

[10] W. Lei, X. Zhao, C. Liang, H. Wang, X. Li, M. Jiang, X. Li, F. He, Y. Sun, G. Lu, H. Cai, Interface-Strengthened Ru-Based Electrocatalyst for High-Efficiency Proton Exchange Membrane Water Electrolysis at Industrial-Level Current Density, Materials, 2024.

[11] X.-Y. Zhang, H. Yin, C.-C. Dang, H. Nie, Z.-X. Huang, S.-H. Zheng, M. Du, Z.-Y. Gu, J.-M. Cao, X.-L. Wu, Unlocking Enhanced Catalysis Stability in Acidic Oxygen Evolution: Structural Insights for PEM Applications under High-Current Density, Angewandte Chemie International Edition, (2025) e202425569.

[12] L. Deng, S.-F. Hung, Z.-Y. Lin, Y. Zhang, C. Zhang, Y. Hao, S. Liu, C.-H. Kuo, H.-Y. Chen, J. Peng, J. Wang, S. Peng, Valence Oscillation of Ru Active Sites for Efficient and Robust Acidic Water Oxidation, Advanced Materials, 35 (2023) 2305939.

[13] Z. Shi, J. Li, Y. Wang, S. Liu, J. Zhu, J. Yang, X. Wang, J. Ni, Z. Jiang, L. Zhang, Y. Wang, C. Liu, W. Xing, J. Ge, Customized Reaction Route for Ruthenium Oxide towards Stabilized Water Oxidation in High-Performance PEM Electrolyzers, Nature Communications, 14 (2023) 843.

[14] Y. Xue, J. Zhao, L. Huang, Y.-R. Lu, A. Malek, G. Gao, Z. Zhuang, D. Wang, C.T. Yavuz, X. Lu, Stabilizing Ruthenium Dioxide with Cation-Anchored Sulfate for Durable Oxygen Evolution in Proton-Exchange Membrane Water Electrolyzers, Nature Communications, 14 (2023) 8093.

[15] S. Zhao, Q. Dang, A. Cao, M.G. Sendeku, H. Liu, J. Peng, Y. Fan, H. Li, F. Wang, Y. Kuang, X. Sun, Hydroxylation Strategy Enables Ru–Mn Oxide for Stable Proton Exchange Membrane Water Electrolysis under 1 A cm^–2^, ACS Nano, (2025).

[16] J. Chen, Y. Ma, C. Cheng, T. Huang, R. Luo, J. Xu, X. Wang, T. Jiang, H. Liu, S. Liu, T. Huang, L. Zhang, W. Chen, Cobalt-Doped Ru@RuO_2_ Core–Shell Heterostructure for Efficient Acidic Water Oxidation in Low-Ru-Loading Proton Exchange Membrane Water Electrolyzers, Journal of the American Chemical Society, (2025).

[17] L. Deng, H. Chen, S.-F. Hung, Y. Zhang, H. Yu, H.-Y. Chen, L. Li, S. Peng, Lewis Acid-Mediated Interfacial Water Supply for Sustainable Proton Exchange Membrane Water Electrolysis, Journal of the American Chemical Society, 146 (2024) 35438-35448.

[18] C. Zhou, L. Li, Z. Dong, F. Lv, H. Guo, K. Wang, M. Li, Z. Qian, N. Ye, Z. Lin, M. Luo, S. Guo, Pinning Effect of Lattice Pb Suppressing Lattice Oxygen Reactivity of Pb-RuO_2_ Enables Stable Industrial-Level Electrolysis, Nature Communications, 15 (2024) 9774.

[19] Y. Shen, X.-L. Zhang, M.-R. Qu, J. Ma, S. Zhu, Y.-L. Min, M.-R. Gao, S.-H. Yu, Cr Dopant Mediates Hydroxyl Spillover on RuO_2_ for High-Efficiency Proton Exchange Membrane Electrolysis, Nature Communications, 15 (2024) 7861.

[20] J. Zhang, X. Fu, S. Kwon, K. Chen, X. Liu, J. Yang, H. Sun, Y. Wang, T. Uchiyama, Y. Uchimoto, S. Li, Y. Li, X. Fan, G. Chen, F. Xia, J. Wu, Y. Li, Q. Yue, L. Qiao, D. Su, H. Zhou, W.A. Goddard, Y. Kang, Tantalum-Stabilized Ruthenium Oxide Electrocatalysts for Industrial Water Electrolysis, Science, 387 (2025) 48-55.

[21] C. Hu, K. Yue, J. Han, X. Liu, L. Liu, Q. Liu, Q. Kong, C.-W. Pao, Z. Hu, K. Suenaga, D. Su, Q. Zhang, X. Wang, Y. Tan, X. Huang, Misoriented High-Entropy Iridium Ruthenium Oxide for Acidic Water Splitting, Science Advances, 9 (2023).

[22] L. Li, G. Zhang, C. Zhou, F. Lv, Y. Tan, Y. Han, H. Luo, D. Wang, Y. Liu, C. Shang, L. Zeng, Q. Huang, R. Zeng, N. Ye, M. Luo, S. Guo, Lanthanide-Regulating Ru-O Covalency Optimizes Acidic Oxygen Evolution Electrocatalysis, Nature Communications, 15 (2024) 4974.
